# Post-translational modifications in ferroptosis: mechanisms and therapeutic potential

**DOI:** 10.7150/ijbs.120624

**Published:** 2026-01-22

**Authors:** Xiaoting Xie, Qianghu Pang, Lianxiang Luo

**Affiliations:** School of Ocean and Tropical Medicine. Guangdong Medical University, Zhanjiang, Guangdong, 524023, China.

**Keywords:** ferroptosis, post-translational modifications, molecular regulation, phosphorylation, ubiquitination

## Abstract

Ferroptosis has been demonstrated to play pivotal roles in a spectrum of pathological processes, including multi-organ dysfunction, retinal degeneration, neurodegenerative disorders, autoimmune diseases, and tumorigenesis. Notably, its pivotal role in counteracting cancer drug resistance positions ferroptosis as a promising therapeutic target. The precise regulation of this cell death pathway is fundamentally dependent on the functional orchestration of associated proteins, where subtle modifications can exert profound effects on ferroptotic progression. Post-translational modifications (PTMs) serve as sophisticated molecular switches that dynamically regulate protein structure, activity, subcellular localization, and functional interactions through covalent attachment of biochemical groups or regulatory subunits. These modifications - including proteolytic processing, partial degradation, or complete protein turnover - significantly expand the functional repertoire of the proteome, thereby exerting crucial regulatory control over cellular survival decisions. This comprehensive review systematically examines the intricate crosstalk between ferroptosis and major PTM pathways, with particular emphasis on ubiquitination, phosphorylation, acetylation, SUMOylation, methylation, oxidative modifications, glycosylation, S-nitrosylation, lactylation, and lipidation. Through critical analysis of current research advances, we elucidate the mechanistic basis by which PTMs modulate ferroptotic pathways and discuss their therapeutic implications. Furthermore, we provide prospective insights into emerging research directions and potential clinical applications targeting PTM-mediated ferroptosis regulation.

## Introduction

Ferroptosis is a form of non-apoptotic cell death characterized by iron-dependent Lipid peroxidation (LPO) of cellular membranes^1^. The onset of ferroptosis is primarily attributed to the effects of ferroptosis-promoting factors or prerequisites outweighing the cellular antioxidant mechanisms and defensive systems against ferroptosis. From a mechanistic standpoint, factors that induce ferroptosis can exert direct or indirect effects on the phospholipid hydroperoxidase, glutathione peroxidase 4 (GPX4), via multiple pathways. This results in a diminished intracellular antioxidant capacity and an accumulation of lipid-derived reactive oxygen species (ROS), subsequently leading to membrane disruption and culminating in cellular demise^2^. Recent studies have indeed demonstrated that ferroptosis is closely associated with the pathophysiological processes of numerous diseases, including autoimmune diseases, neurological disorders, ischemia-reperfusion injury, renal damage, hematological diseases, and particularly in the context of cancer and its resistance to chemotherapy, which holds significant potential^3^. Ferroptosis underlies pollutant-elicited organ injury; its pharmacological modulation (Nrf2 activators, iron chelators) constitutes an emerging therapeutic axis against environmental health hazards^4^. Numerous pathways can modulate ferroptosis, including metabolic pathways and degradation pathways, which can play a double-edged sword role in disease therapy. Similarly, an increasing number of studies have found that ferroptosis can also be regulated in the process of epigenetics, including histone PTMs, DNA methylation, non-coding RNA regulation, and PTMs^5^. Epigenetic alterations have garnered considerable attention due to their significance in regulating biological processes and their potential as therapeutic targets. Among these, PTMs drive the dynamic dysregulation of transcription or translation, and more importantly, key PTMs modulating ferroptosis have been identified as potential targets for cancer therapy. The functional involvement of two protein subunits, solute carrier family 7 member 11 (SLC7A11) and GPX4, in ferroptosis resistance has been extensively studied in recent years. Studies have indicated that PTMs play a positive role in the treatment of diseases by modifying ferroptosis-related proteins in liver cancer^6^.

PTMs represent a chemical modification that targets the structural diversity and functional specificity of the proteome^7^. PTMs regulate gene expression post-transcriptionally, thereby modulating protein activity, structure, and function^8^. Research indicates that the dynamic interplay between protein degradation pathways and PTMs offers potential therapeutic targets to modulate the onset of ferroptosis^9^. The extensive repertoire of PTMs is instrumental in modulating the mechanisms of ferroptosis within cellular and organelle contexts^10^. Ubiquitination of specific lysine residues on proteins associated with ferroptosis can hinder the resistance to ferroptosis conferred by Ferroptosis Suppressor Protein 1 (FSP1)^11^. Deubiquitinating enzymes contribute to the recycling of ubiquitin by degrading ubiquitin polymers, thus playing a pivotal role in the regulation of ferroptotic mechanisms. A dynamic regulatory mechanism modulating ferroptosis involves the complex interplay between SUMOylation and ubiquitination, which can influence cellular ferroptosis by either upregulating or downregulating ubiquitination^9^. Phosphorylation, a widely occurring covalent modification among PTMs, exerts a substantial influence on the modulation of ferroptotic processes through its covalent modifications on proteins such as GPX4^12^, within the Hippo signaling pathway^13^,^14^, the AMP-activated protein kinase (AMPK) pathway^15^, and in ferroptosis metabolism^16^. The dynamic regulation by protein kinases and protein phosphatases further establishes phosphorylation as a crucial regulatory mechanism in ferroptosis. Lipidation directs proteins to cellular organelles and the plasma membrane, augmenting their membrane affinity. This includes four non-exclusive types of lipidation: glycosyl phosphatidylinositol (GPI) anchoring, N-myristoylation^17^, S-palmitoylation^18^, and S-prenylation, which can occur singularly or in combination on the same protein and are potentially linked to ferroptosis. Acetylation, encompassing proteins such as tumor protein p53 (TP53), ALOX12, HMGB1, HSPA5, and histones, is implicated in the modulation of ferroptosis, either by promoting or inhibiting the process^9^. Methylation, another PTM that targets both histone and non-histone proteins, is integral to cellular function and has been found to have a connection with ferroptosis^9^. High oxidation peroxiredoxin 3 (PRDX3) has been identified as a potential biomarker for ferroptosis in chronic liver disease^19^. Oxidative modifications which can lead to alterations in protein function, have garnered our interest. Glycosylation, one of the major PTMs, has not been extensively studied in its association with ferroptosis, but research has found that glycosylation of the heavy chain subunit of system Xc- can induce the susceptibility of pancreatic ductal adenocarcinoma to ferroptosis^20^. S-nitrosylation is a reversible PTM that serves to stabilize proteins, regulate gene expression, and provide a nitric oxide (NO) donor in cells. Ferroptosis in non-small cell lung cancer (NSCLC) can be achieved by reducing intracellular glutathione (GSH) levels via NO donors, elevating malondialdehyde (MDA) levels, inhibiting the expression of SLC7A11/GSH proteins, and negatively regulating the JAK2/STAT3 pathway^21^. Lysine lactylation, derived from lactic acid, is a novel PTM that provides insights into the function of lactic acid and its role in infection and inflammation, and here we discuss its connection with ferroptosis^22^.

PTMs, each with their unique characteristics, are not entirely independent of each other. Proteins can be subject to not only a single modification but also to two or more modifications simultaneously. Therefore, there exists an interconnected and complex network system of potential PTMs. In summary, there is a close relationship between ferroptosis and the PTMs network system. Consequently, studying PTMs in the context of ferroptosis holds promising prospects for the development of drugs with high specificity, selectivity, and low toxicity for the treatment of various diseases and cancers in the future.

## 1. The Process of Ferroptosis and its Regulatory Mechanisms

### 1.1. The Antioxidant Basis of Ferroptosis

Cells possess an integrated array of antioxidant defense mechanisms that rigorously prevent excessive accumulation of lipid peroxides and the ensuing initiation of ferroptosis. Recent years have seen the discovery of four GPX4-independent systems that effectively inhibit ferroptosis. FSP1/coenzyme Q10, dihydroorotate dehydrogenase (DHODH), GTP cyclohydrolase 1 (GCH1)/tetrahydrobiopterin (BH4), and MBOAT1/2-MUFA each independently inhibit ferroptosis apart from GPX4. Below, we will provide a brief overview of these common antioxidant pathways against ferroptosis.

#### 1.1.1. SLC7A11-GSH-GPX4

GPX4 is the most important protein against ferroptosis; it is a selenocysteine-containing protein, and selenium can enhance the cell's antioxidant capacity during ferroptosis. Consequently, GPX4 utilizes GSH as a cofactor to convert lipid peroxides into non-toxic metabolites, thus preventing the buildup of lipid peroxides. The antioxidant GSH is a tripeptide composed of glutamate, cysteine, and glycine. Due to the limited concentration of cysteine in cells, cysteine is considered the rate-limiting precursor for GSH synthesis. Cystine, in its oxidized state, is transported into the cell via the system Xc- and promptly reduced to cysteine intracellularly. System Xc- is a heterodimeric protein complex composed of the regulatory subunit solute carrier family 3 member 2 (SLC3A2) and the catalytic SLC7A11. This complex facilitates the exchange of extracellular cystine and intracellular glutamate on the plasma membrane. Cystine in the cell is reduced to cysteine, which is essential for the production of GSH. GPX4 uses GSH to eliminate the formation of phospholipid hydroperoxides (PLOOH), thus inhibiting ferroptosis^23^, ^24^.

GPX4 constitutes the linchpin of the antioxidant machinery that counteracts ferroptosis; abundant evidence now indicates that pharmacological modulation of GPX4 exerts a Janus-faced influence on ferroptosis-dependent therapeutic outcomes across a spectrum of diseases. PTMs enhance the functional versatility of the proteome through the addition of functional groups or covalently attaching regulatory subunits to proteins, proteolytic cleavage of proteins, or the degradation of entire proteins. GPX4 inactivation, stemming from mutations or covalent modifications of the active site selenocysteine, impairs its function in halting the complex lipid oxidation cascade, thus augmenting ferroptosis^25^. For tumor, DMOCPTL, a derivative of parthenolide (PTL), has been identified as a potential therapeutic agent targeting triple-negative breast cancer (TNBC) cells. It induces ferroptosis by ubiquitinating GPX4^26^. Creatine kinase B (CKB) can induce ferroptosis by ubiquitinating GPX4. Specifically, CKB phosphorylates GPX4 at the Ser104 residue, thereby preventing its degradation and counteracting ferroptosis in hepatocellular carcinoma (HCC) cells, ultimately promoting tumor growth^12^. In Alzheimer's disease, SIRT1 depletion attenuates GPX4 deacetylation, suppressing its peroxidase activity, thereby potentiating lipid peroxidation and exacerbating amyloid-β-triggered neurotoxicity. During myocardial ischemia-reperfusion(I/R), AMPK-dependent phosphorylation of GPX4 at Ser104 inactivates the enzyme, precipitating cardiomyocyte ferroptosis^27^. In non-alcoholic fatty liver disease (NAFLD), the Tibetan remedy Huagan Tongluo Formula (HGTLF) triggers ferroptosis of hepatic stellate cells via ubiquitin-dependent degradation of GPX4, consequently ameliorating hepatic fibrosis^28^. These findings offer novel perspectives on the modulation of ferroptosis via the SLC7A11-GSH-GPX4 axis. Nevertheless, additional studies are required to investigate whether different PTMs can modify the proteins in this pathway.

#### 1.1.2. NADPH-FSP1-CoQ10

FSP1 has been identified as a second ferroptosis-inhibitory system when combined with extracellular ubiquinone or exogenous vitamins K and NAD(P)H/H+ as electron donors, all of which exhibit significant radical trapping antioxidant (RTA) activity and can effectively prevent LPO. FSP1 is localized on the plasma membrane, where it can produce CoQ10 (also known as ubiquinone), an endogenous electron carrier, and the reduced form of vitamin K. FSP1, an NAD(P)H-dependent oxidoreductase, catalyzes the reduction of CoQ10 to its reduced form, CoQ10-H_2_, thereby scavenging LPO radicals and inhibiting LPO and the initiation of ferroptosis^29^. Apart from CoQ10, FSP1 is also capable of regenerating reduced vitamin K, an additional endogenous RTA that can restrict LPO and ferroptosis^30^. Several investigations have demonstrated that the inhibition of the FSP1-CoQ axis can effectively reverse radioresistance^31^. Research has found that modifications to FSP1 through PTMs can also affect ferroptosis. Since the N-terminus of FSP1 contains a typical myristoylation motif, and myristoylation is a lipid modification that promotes the binding of target proteins to the cell membrane, the myristoylation of the N-terminus of the FSP1 protein facilitates the localization of FSP1 to the plasma membrane, which is crucial for its ferroptosis-inhibiting activity^17^, ^32^, ^33^. Acyl-CoA Synthetase Long-Chain Family Member 1 (ACSL1) can reduce the level of lipid oxidation and increase the resistance to ferroptosis in cells. Mechanistically, ACSL1 increases the N-myristoylation of FSP1, thereby inhibiting its degradation and translocation to the cell membrane. The increase in myristoylated FSP1 functionally counteracts cell ferroptosis induced by oxidative stress^34^. Sorafenib triggers ferroptosis in hepatocellular carcinoma by facilitating TRIM54-mediated ubiquitination and degradation of FSP1^35^. RNF126 interacts with FSP1 and ubiquitinates FSP1 at the 4KR-2 site, acting as an anti-ferroptotic gene. Furthermore, the lack of RNF126 diminishes the subcellular localization of FSP1 at the plasma membrane, resulting in an elevated CoQ/CoQH2 ratio in G3-medulloblastoma^36^. Exogenous nicotinamide adenine dinucleotide phosphate (NADPH) Interacts with N-myristoyltransferase 2, leading to the upregulation of N-myristoylated FSP1. This interaction facilitates the membrane localization of FSP1 and augments its resistance to ferroptosis^37^. The perspective of resisting ferroptosis through N-myristoylation and ubiquitination of FSP1 provides a promising avenue for the regulation of ferroptosis mechanisms; however, we still require a substantial amount of research to expand and validate its therapeutic efficacy in actual clinical settings for diseases.

#### 1.1.3. GCH1-BH4

GCH1-BH4 is a novel pathway for regulating ferroptosis identified through metabolic-focused CRISPR-Cas9 gene screening and genome-wide dCas9-based activation screening (CRISPRa). Overexpression of GCH1 not only eliminates LPO but also nearly completely blocks the occurrence of ferroptosis. Tetrahydrobiopterin (BH4), a constituent of the antioxidant system, is implicated in the metabolism of NO, neurotransmitters, and aromatic amino acids. GCH1 serves as the rate-limiting enzyme in BH4 synthesis^38^. By synthesizing BH4 via GCH1 and subsequently reducing dihydrobiopterin (BH2) to BH4 through dihydrofolate reductase (DHFR), BH4 functions as an endogenous anti-ferroptotic RTA^39^. Additionally, studies have shown that cells with overexpressed GCH1 have phospholipids protected by two polyunsaturated fatty acid (PUFA) chains, thereby enriching the reduced CoQ10 levels post-ferroptosis induction, such as under IKE treatment^40^. The GCH1-BH4 pathway, as an endogenous antioxidant pathway, inhibits ferroptosis through mechanisms independent of the GPX4/GSH system. Current ferroptosis research on the GCH1-BH4 axis remains confined to “abundance/activity” metrics (transcriptional control, protein level fluctuations, BH4 synthesis rate), leaving its post-translational landscape virtually unexplored. Systematic mapping of GCH1 phosphorylation, ubiquitination, or acetylation, together with oxidative/nitrosative modifications of BH4, represents an urgent and tractable knowledge gap.

#### 1.1.4. DHODH-CoQH_2_

DHODH operates in parallel with mitochondrial GPX4 (but independently of cytosolic GPX4 or FSP1), and is located on the outer surface of the mitochondrial inner membrane (IMM), where it intercepts LPO radicals by reducing CoQ10 to CoQH_2_, thereby inhibiting ferroptosis within the IMM^41^. In overcoming cancer's resistance to chemotherapy, research indicates that the depletion of proline-rich protein 11 (PRR11) markedly enhances the sensitivity of glioblastoma multiforme (GBM) cells to temozolomide (TMZ) through the induction of ferroptosis. Mechanistically, PRR11 directly interacts with and stabilizes DHODH, conferring resistance to glioma ferroptosis in a DHODH-dependent manner both *in vivo* and *in vitro*. Moreover, PRR11 inhibits the recruitment of the E3 ubiquitin ligase HERC4 and prevents the polyubiquitination and degradation of DHODH at the K306 site, thereby maintaining DHODH protein stability. Notably, the downregulation of PRR11 augments LPO and disrupts DHODH-regulated mitochondrial morphology, consequently facilitating ferroptosis and increasing TMZ chemosensitivity in GBM cells^42^. Lysyl oxidase-like 3 (LOXL3) depletion significantly sensitizes liver cancer cells to oxaliplatin (OXA) by inducing ferroptosis. Chemotherapy-activated EGFR signaling drives the interaction between LOXL3 and TOM20, leading to the hijacking of LOXL3 to mitochondria, where the lysyl oxidase activity of LOXL3 is enhanced through phosphorylation at the S704 site. Metabolic adenylate kinase 2 (AK2) directly phosphorylates LOXL3-S704. Phosphorylated LOXL3-S704 targets DHODH and stabilizes it by preventing its ubiquitin-mediated proteasomal degradation. K344-deubiquitinated DHODH accumulates in mitochondria, thereby inhibiting chemotherapy-induced mitochondrial ferroptosis. In a late-stage liver cancer mouse model, the combination of low-dose OXA with the DHODH inhibitor leflunomide effectively inhibits liver cancer progression by inducing ferroptosis, increasing chemotherapy sensitivity, and reducing chemotherapy toxicity^43^. This all demonstrates the potential of the DHODH-CoQH_2_ pathway, and targeting this pathway may be able to regulate the resistance of cancer cells to ferroptosis inducers.

#### 1.1.5. MBOAT1/2-MUFA

Increasing evidence suggests that steroid hormones are central regulators of several ferroptosis regulatory systems. The expression of membrane-bound O-acyltransferase domain containing 1 and 2 (MBOAT1/2), driven by sex hormones, can enable cancer cells to escape ferroptosis. MBOAT1 and MBOAT2 are transcriptionally upregulated by the sex hormone receptors estrogen receptor (ER) and androgen receptor (AR), respectively. The combination of ER or AR antagonists with ferroptosis inducers can significantly inhibit the growth of ER+ breast cancer and AR+ prostate cancer, even when the tumors are resistant to monotherapy with hormone treatment alone^44^. Phospholipids laden with polyunsaturated fatty acids (PUFAs) are prone to LPO. Conversely, MBOAT2 preferentially integrates monounsaturated fatty acids (MUFAs) into phospholipids, indicating that MBOAT2 might mitigate ferroptosis by competitively lowering the PUFA levels in phospholipids. In fact, lipidomics analysis has shown that overexpression of MBOAT2 specifically increases phosphatidylethanolamine (PE) containing MUFAs while decreasing PUFA-PE, thereby enhancing resistance to ferroptosis^45^, ^46^. Although no PTMs of MBOAT1/2 have yet been reported, the pivotal MUFA-synthesizing enzyme SCD1 and its upstream lipoylation machinery are known to be PTM-regulated. This suggests that manipulation of PTMs in these peripheral enzymes may offer an indirect yet potent strategy to modulate the ferroptosis-suppressive function of the MBOAT1/2-MUFA axis.

### 1.2. The Core Mechanism of Ferroptosis and Related Metabolism[Figure 1]

ROS are produced by chemical reactions between lipids, oxygen, and iron. Phospholipids containing polyunsaturated fatty acids (PUFA-PLs; fatty acids with multiple double bonds) are the main substrates in the process of ferroptosis. Acyl-CoA synthetase long-chain family member 4 (ACSL4) activates free polyunsaturated fatty acids (PUFAs), particularly arachidonic acid (AA) and adrenic acid (AdA), into PUFA-CoA. Lysophosphatidylcholine acyltransferase 3 (LPCAT3) then incorporates these acyl-CoAs into phosphatidylcholine/ethanolamine, generating PUFA-PLs that are enriched in the cell membrane and mitochondrial membrane, serving as a reservoir for lipid peroxidation (Dai *et al.*, 2024). Subsequently, lipoxygenases from the linoleate/arachidonate lipoxygenase family (ALOX) catalyze the formation of lipid hydroperoxides (PLOOH) from PUFA-PLs, thereby promoting ferroptosis. Cytochrome P450 oxidoreductase (POR) is also a redox enzyme that, independent of ALOX, can promote the peroxidation of polyunsaturated phospholipids. It transfers electrons from NADPH to molecular oxygen, forming H_2_O_2_, which induces cells to undergo Fenton reactions with Fe^2+^, promoting the peroxidation of PUFAs, thereby inducing ferroptosis^47^. However, the development of ferroptosis associated with PUFAs can be effectively blocked by the production and stimulation of MUFAs. This blockage is facilitated by acyl-CoA synthetase long-chain family member 3 (ACSL3) or stearoyl-CoA desaturase (SCD/SCD1)^48^. Conversely, attenuation of IR/SREBP axis-mediated MUFA synthesis facilitates ferroptosis in HCC^49^.

If the aforementioned processes are considered as the establishment of a “fuel depot” for ferroptosis, then iron acts as an “igniter” in this context. The role of iron in ferroptosis is “trifunctional”: i) It serves as a catalyst for the Fenton reaction, directly generating the hydroxyl radicals required for lipid peroxidation; ii) It acts as a cofactor for iron-containing enzymes such as ALOXs and cytochrome P450, continuously producing lipid peroxides; iii) It provides a continuous supply of free iron through ferritinophagy, thereby forming a positive feedback loop that amplifies the death signal. Extracellular Fe^3+^ in the body binds to ferroportin (FPN) on the cell membrane, forms a complex with the membrane protein TF receptor 1 (TFRC), and is transported into the cell through endocytosis^50^. Upon cellular uptake, Fe^3+^ is reduced to Fe^2+^ by the six-transmembrane epithelial antigen of the prostate 3 (STEAP3) within the endosome, subsequently being translocated into the cytoplasm via the divalent metal transporter 1 (DMT1) protein^51^. Fe^2+^ in the cytoplasm and mitochondria establishes a labile iron pool (LIP), and through the mediation of Fenton reaction-induced LPO, it shows oxidative activity and induces ferroptosis. The iron-catalyzed non-enzymatic Fenton chain reaction could be pivotal to ferroptosis. Upon inhibition of GPX4, PLOOHs may endure for extended durations, and the onset of the Fenton reaction can swiftly escalate the levels of PLOOHs. PLOOHs can react with Fe^2+^, producing radical PLO• and PLO•, respectively. These radicals react with PUFA-PLs, further propagating the production of PLOOHs and promoting the occurrence of ferroptosis. Furthermore, research has discovered that the process of ferritin autophagy mediated by nuclear receptor coactivator 4 (NCOA4) can degrade ferritin, releasing the iron stored within it into the LIP, enhancing the availability of iron within cells, and thereby promoting LPO-driven ferroptosis^52^, ^53^. Moreover, hepcidin binds to FPN1 on the cell membrane surface, thereby promoting the endocytosis and degradation of FPN1. This process inhibits iron absorption by intestinal epithelial cells and iron recycling from aged erythrocytes by macrophages.

The core mechanism of ferroptosis is implicated in the pathogenesis and progression of many diseases. For instance, the immediate surge of free Fe²⁺ during reperfusion drives lipid peroxidation mediated by ACSL4/Alox15, and the peroxidation of mitochondrial cardiolipin leads to the opening of the mitochondrial permeability transition pore (mPTP), resulting in ferroptosis of cardiomyocytes. Blocking iron uptake or lipid oxidation by using TFR1 antibodies, Alox15 inhibitors, or GPX4 activators within 5-30 min can reduce infarction by 40%^54^.

Amino acid metabolic processes constitute a significant determinant in the induction of ferroptosis. When discussing SLC7A11-GSH-GPX4, we mentioned that cysteine (Cys), an important synthetic rate-limiting precursor of GSH, is a crucial cofactor for GPX4. During the progression of aortic aneurysms, downregulation of GPX4 and SLC7A11, coupled with the accumulation of LPO, leads to ferroptosis in vascular smooth muscle cells (VSMCs) and endothelial cells. This process triggers medial degeneration and the formation of dissections^55^. The exogenous sources of Cys are through the direct absorption of Cys by excitatory amino acid transporter 3 (EAAT3) and alanine/serine/cysteine/threonine transporter 1, or through the intake of cystine via SLC7A11 and SLC3A2, which is then converted to Cys in the cytoplasm through the catalyzation of glutamate-cysteine ligase (GCL). The endogenous source of Cys involves the transsulfuration pathway, which includes the conversion of methionine to S-adenosylmethionine by the rate-limiting enzyme methionine adenosyltransferase 2A (MAT2A), followed by a series of biochemical reactions, ultimately forming Cys^56^. However, the level of Cys within cells can also be suppressed. Cysteine dioxygenase (CDO1) facilitates the transformation of Cys into taurine. In this process, CDO1 competes with GCL for Cys, effectively redirecting Cys that would otherwise be utilized for the synthesis of GSH towards taurine biosynthesis^57^. The suppression or functional inactivation of CDO1 facilitates the repletion of intracellular GSH levels, mitigating the accumulation of reactive ROS and LPO. This, in turn, enhances cellular resilience against ferroptosis and fosters tumor growth^58^, ^59^. As another precursor for GSH synthesis, glutamate can aid in the formation of GSH and NADPH. Extracellular glutamine (Gln) enters the cell via SLC1A5 (ASCT2) and is converted to glutamate (Glu) under the catalysis of glutaminase (GLS), which is used with Cys and Gly to produce GSH. The synthesized GSH and NADPH serve to sustain redox homeostasis^60^, ^61^.

Lipid metabolism, iron metabolism, and amino acid metabolism complement and influence each other in the process of ferroptosis, interweaving into the metabolic network of ferroptosis. Among these, PTMs of ferroptosis related proteins hold significant potential for regulating cellular ferroptosis. For instance, protein kinase C beta II (PKCβII) promotes the generation of PUFA-PLs by phosphorylating ACSL4. This process in turn increases the phosphorylation of NCOA4, leading to enhanced ferritinophagy and the release of more free iron. Consequently, the lipid peroxidation cascade is amplified^16^. AMPK phosphorylates BECN1, which leads to an increased formation of the BECN1-SLC7A11 complex. This results in reduced cystine uptake, impaired synthesis of GSH, and inactivation of GPX4, thereby further inducing ferroptosis^62^. In the treatment of liver cancer, Erastin can trigger de-O-GlcNAcylation of TFRC at serine 687 (Ser687), thereby reducing the binding of the ubiquitin E3 ligase membrane-associated RING-CH8 (MARCH8) and decreasing polyubiquitination on lysine 665 (Lys665), which enhances the stability of TFRC that favors the accumulation of labile iron and promotes the occurrence of ferroptosis^63^. The anti-proliferative effects of small molecule ferroptosis modulators, particularly class I histone deacetylase (HDAC) inhibitors, is markedly reliant on the ferroptosis pathway. The HDAC inhibitor HL-5s demonstrates significant antagonistic efficacy toward class I HDACs, with a particular focus on HDAC1. Mechanistically, HL-5s increases YB-1 acetylation, inhibits the nuclear factor erythroid 2-related factor 2 (Nrf2)/heme oxygenase-1 (HO-1) signaling pathway, elevates Fe^2+^ levels, and generates ROS through the Fenton reaction, ultimately promoting the production of LPO, which triggers ferroptosis^64^. The JAK-STAT signaling pathway, which is related to inflammatory signaling, promotes the expression of hepcidin by activating STAT3, thereby inhibiting iron efflux^65^. In zebrafish exposed to high iron levels, IL-22 triggers STAT3 phosphorylation, leading to increased hepcidin expression and hepatic iron accumulation. This results in iron overload-induced ferroptosis in the liver^66^. Some scholars have also confirmed in animal experiments that ferroptosis can be precisely “switched on and off” within minutes through reversible PTMs such as phosphorylation, ubiquitination, or deacetylation. In a mouse model, AAV9-SIRT3 mediated deacetylation of GPX4 reduced the infarct size in I/R injury by 25%. In patient-derived xenograft (PDX) models, the USP14 inhibitor IU1, when used in combination with cisplatin, increased ubiquitination of GPX4, thereby inducing ferroptosis in more tumor cells and reducing tumor volume. These studies have achieved quantifiable therapeutic effects at the mouse/rat level and may provide new targets for drug development^67^. We will provide a more detailed discussion on different PTMs in the subsequent sections.

## 2. The Regulatory Role of PTMs in Ferroptosis

PTMs refer to the collective term for specific chemical modifications at the protein level. After amino acids within the cell are transcribed into mRNA and translated into inactive proteins, these proteins are endowed with activity and functionality through covalent modifications and linkages with different functional groups or proteins^68^, regulatory hydrolytic cleavage of protein subunits, or partial or complete degradation. This process leads to differentiation at various structural levels of proteins, diversification in function, and enrichment of the proteome within the cell. The occurrence of PTMs is of significant importance for the normal operation of cell proliferation, death, localization, and function^9^. PTMs can occur at any stage of the protein life cycle, and they are typically mediated by specific enzymatic activities. Therefore, depending on the nature of different PTMs, these processes are reversible and function to modulate ferroptosis, thereby affecting the sensitivity of diverse pathophysiological mechanisms^9^. The dysregulation of PTMs may lead to abnormal expression of protein characteristics, even transformation into malignant phenotypes, further inducing the occurrence and progression of diseases^68^. The interaction between PTMs and protein degradation mechanisms^69^ also provides multiple approaches and pathways for the intervention of ferroptosis.[**Error! Not a valid bookmark self-reference.**]

### 2.1. Ubiquitination

Ubiquitination is the process by which ubiquitin, a polypeptide composed of 76 amino acids, combines with target substrates to form ubiquitin chains. Specific lysine (K) residues are the primary sites for ubiquitination. The sequential cascade of catalytic reactions mediated by E1 ubiquitin-activating enzymes, E2 ubiquitin-conjugating enzymes, and E3 ubiquitin ligases orchestrates the entire ubiquitination process^9^. Ubiquitination is considered a tightly regulated PTM, and Deubiquitinases (DUBs) can reverse the process of ubiquitination^70^, regulating protein stability and degradation. Ubiquitination has now become a key regulatory mechanism in ferroptosis. For example, Overexpression of the E3 ubiquitin ligase TRIM7^71^ leads to the ubiquitination of NCOA4, inhibiting NCOA4-mediated ferroptosis in GBM^72^.

#### 2.1.1. FSP1-Related Ubiquitination

In the early stages of ferroptosis, the ubiquitination and degradation of glutathione S-transferase P1 (GSTP1) mediated by the SMAD-specific E3 ubiquitin ligase 2 (SMURF2) lead to a significant downregulation of GSTP1. SMURF2 primarily promotes the degradation of GSTP1 protein by catalyzing the polyubiquitination of GSTP1 at K121/191/55 sites, thereby promoting ferroptosis^73^. Recent investigations have revealed a strong correlation between K63-linked ubiquitination and the plasma membrane localization of target proteins, as well as the role of FSP1 in ferroptosis inhibition. Specifically, the E3 ubiquitin ligase TRIM21 has been shown to facilitate K63-linked ubiquitination of FSP1 at K322 and K366, which disrupts the plasma membrane localization and ferroptosis resistance of FSP1^9^. In pancreatic cancer, TRIM21 facilitates tumor growth and gemcitabine resistance while inhibiting ferroptosis through the suppression of arachidonic acid metabolism mediated by Microsomal epoxide hydrolase 1 (EPHX1). Bezafibrate can disrupt the interaction between TRIM21 and EPHX1, thereby enhancing the efficacy of gemcitabine and presenting a novel therapeutic strategy for pancreatic cancer^74^. Edaravone, a new ferroptosis inhibitor, stabilizes FSP1 protein levels by inhibiting FSP1 ubiquitination, suppresses ferroptosis, and alleviates doxorubicin (DOX)-induced cardiotoxicity^75^.

#### 2.1.2. SLC7A11-GSH-GPX4 Pathway- Related Ubiquitination

The E3 ubiquitin ligase TRIM25 binds to the ferroptosis inducer N6F11, mediating the K48 polyubiquitination of GPX4. TRIM25 is activated by the ubiquitin-conjugating enzyme (E2) Ubc5 domain, enabling N6F11 to activate the UbcH5b-TRIM25-GPX4 ubiquitination cascade, triggering ferroptosis in cancer cells^76^. The expression of the E3 ubiquitin ligase TRIM3 is significantly reduced in lung adenocarcinoma (LUAD) and lung squamous cell carcinoma (LUSC), which are predominant subtypes of non-small cell lung cancer (NSCLC), but overexpression of TRIM3 can increase ROS levels and LPO in cancer cells, promoting ferroptosis^77^. In melanoma cells, the E3 ubiquitin ligase Nedd4 degrades the voltage-dependent anion channel VDAC2/3 by binding to the PPxY motif of VDAC2/3 through its WW domain. Conversely, Erastin induces ferroptosis by directly binding to VDAC2/3, thereby altering the permeability of the mitochondrial outer membrane and decreasing the rate of NADH oxidation^78^. Nedd4 also mediates the ubiquitination of GPX4, disrupting its function and inducing ferroptosis, elucidating the mechanisms underlying dopaminergic neuronal degeneration in the pathogenesis of Parkinson's disease (PD)^79^. The HECT domain-containing ubiquitin E3 ligase HUWE1, as a negative regulator of ferroptosis, specifically targets the transferrin receptor 1 (TfR1) for ubiquitination and proteasomal degradation, regulating iron metabolism, counteracting abnormal iron accumulation, inhibiting ferroptosis, and potentially mitigating acute liver injury caused by ischemia-reperfusion (I/R)^80^. The E3 ubiquitin ligase RC3H1 stabilizes the GPX4 protein via the cleavage of mucosa-associated lymphoid tissue lymphoma translocation protein 1 (MALT1), thereby exerting an inhibitory effect on ferroptosis. The MALT1 inhibitor MI-2 not only induces ferroptosis in hepatocellular carcinoma cells but also demonstrates synergistic anticancer effects when combined with sorafenib or regorafenib. The crucial role of MALT1 in the ferroptosis pathway underscores its potential as a novel therapeutic target for cancer treatment^81^.

Ubiquitin-specific protease 7 (USP7) stabilizes heterogeneous nuclear ribonucleoprotein A1 (hnRNPA1) via deubiquitination, facilitating the entry of miR-522 from cancer-associated fibroblasts in the tumor microenvironment into exosomes. These exosomes are subsequently activated by cisplatin and paclitaxel, leading to downregulation of ROS levels in gastric cancer cells, inhibition of ferroptosis, and reduced chemotherapy sensitivity^82^. At the same time, studies have found that USP7 promotes tumorigenesis by increasing the levels of stearoyl-CoA desaturase (SCD). The USP7 inhibitor DHPO induces ferroptosis in gastric cancer, and the inhibition of USP7 by DHPO increases the ubiquitination of SCD, accelerating its proteasomal degradation, thereby inhibiting the growth and metastasis of gastric cancer^83^. Ubiquitin-specific protease 8 (USP8) stabilizes GPX4 by removing K48-linked ubiquitination, thereby inhibiting ferroptosis and potentially enhancing cancer immunotherapy^84^. The tumor suppressor BRCA1-associated protein 1 (BAP1) diminishes the occupancy of histone 2A ubiquitination (H2Aub) at the SLC7A11 promoter, thereby downregulating SLC7A11 expression in a deubiquitination-dependent manner. Additionally, BAP1 curtails cystine uptake by repressing SLC7A11 expression, which subsequently escalates LPO and ferroptosis^85^. Highly expressed cytokine signaling 2 inhibitor (SOCS2) promotes ferroptosis by facilitating the ubiquitination and degradation of SLC7A11, where SLC7A11 polyubiquitination mainly occurs in the form of K48-linked ubiquitin chains^86^. The tumor suppressor PCDHB14 promotes the ubiquitination of p65, preventing it from binding to the SLC7A11 promoter, downregulating the levels of SLC7A11 in HCC, and enhancing ferroptosis^87^.

In cholangiocarcinoma (CCA), the expression of SLC7A11 is upregulated due to the increased expression of shank-associated RH domain interacting protein (SHARPIN), a component of the linear ubiquitin chain complex^88^. The inactivation of SLC7A11 due to ubiquitination also induces ferroptosis in NSCLC^89^ and ovarian cancer cells^68^. The downregulation of p21 expression due to p53 ubiquitination subsequently induces ferroptosis in colorectal cancer (CRC) cells by inhibiting GPX4^90^. GPX4 is a well-recognized significant negative regulator of ferroptosis^91^, and DMOCPTL, as the first reported to induce ferroptosis by ubiquitinating GPX4 and downregulating GPX4 levels, supports the treatment of TNBC^26^. The potential mechanism of low back pain caused by intervertebral disc degeneration (IVDD) is partly considered to be ferroptosis. Polydopamine nanoparticles (PDA NPs) colocalize with GPX4 around the mitochondria, inhibit ubiquitin-mediated degradation, downregulate the production of MDA and LPO, and antagonize ferroptosis in nucleus pulposus cells^92^. Sirtuin 3 (SIRT3) is a key regulator of mitochondrial ROS, and it plays a role in promoting the expression of GPX4 to inhibit ferroptosis in gallbladder cancer. Ubiquitin-specific protease 11 (USP11) can directly bind to Sirt3 and deubiquitinate Sirt3 to stabilize Sirt3, inhibiting its degradation. The occurrence of IVDD downregulates the level of Sirt3, upregulates the level of oxidative stress, induces ferroptosis, and exacerbates patient pain^93^. The androgen receptor (AR) enhances the drug resistance of GBM to TMZ, and curcumin analog ALZ003-mediated AR ubiquitination causes astrocytoma cells to undergo ferroptosis, potentially opening up new avenues for the treatment of TMZ-resistant GBM^94^.[**Table 2**]

### 2.2. SUMOylation

SUMOylation, a PTM characterized by the covalent attachment of small ubiquitin-like modifier (SUMO) proteins to target proteins, plays a crucial role in diverse cellular processes and serves as a vital cellular mechanism for stress response. Importantly, SUMOylation is a reversible and dynamic process^68^. It conjugates to the lysine (Lys) residues of substrate proteins during the PTM process. There are five SUMO isoforms in mammalian cells, namely SUMO1, SUMO2, SUMO3, SUMO4, and SUMO5. SUMO molecules are covalently attached to target proteins via an enzymatic cascade analogous to the ubiquitin cascade, which entails the sequential involvement of E1, E2, and E3 enzymes. Conversely, SUMO-specific proteases, SENPs (SENP1, SENP2, SENP3, SENP5, SENP6, and SENP7), catalyze de-SUMOylation and precursor processing. The balance of cellular SUMOylation is determined by the restrictive SUMO precursors and mature SUMO isoforms, as well as the SENP family^95^.

Based on their distinct subcellular localizations and substrates, SENPs are categorized into three distinct classes: The first category comprises SENP1 and SENP2, which are localized to the nuclear pore or PML nuclear bodies in interphase cells and exhibit broad isopeptidase activity against SUMO1, SUMO2, and SUMO3; The second category encompasses SENP3 and SENP5, which are localized to the nucleolus and have high specificity for SUMO2 and SUMO3; the third category includes SENP6 and SENP7, which are localized to the nucleoplasm and are relatively inclined to cleave SUMO2 and SUMO3 chains. SENP6 and SENP7 have minimal involvement in precursor processing; in contrast, SENP1, SENP2, and SENP5 participate in the SUMO maturation process^95^. Emerging research indicates that SUMOylation regulate ferroptosis in cancer. The upregulation of ROS levels, particularly H_2_O_2_, and the occurrence of LPO are typical characteristics of ferroptosis. In various cancers, the overactivation of the SUMO pathway is considered a selective advantage in combating stress. It is hypothesized that this is a mechanism by which cancer cells enhance their tolerance to ROS-induced upregulation to avoid death. The modulation of ferroptosis-related proteins via SUMOylation has emerged as a potential therapeutic strategy. Even in extracts of certain traditional Chinese herbal components, such as ginkgolic acid (GA) induces ferroptosis in hepatic stellate cells by targeting the SUMOylation marker SUMO1 activating enzyme subunit 1 (SAE1), thereby exerting anti-liver fibrosis effects^96^.

#### 2.2.1. SLC7A11-GSH-GPX4 Pathway- Related SUMOylation

A series of sites susceptible to ferroptosis are targets of SUMOylation, including the central ferroptosis inhibitor GPX4, which inhibits the peroxidation of membrane phospholipids and participates in ferroptosis after SUMO modification. Nrf2, an antioxidant transcription factor intricately linked to ferroptosis, undergoes ubiquitination at the conserved lysine residue K110. SUMOylation can enhance Nrf2's activity in scavenging ROS, ultimately enhancing the tolerance of liver cancer cells to oxidative stress^95^. Overexpression of SENP1 attenuates ferroptosis triggered by Erastin or cisplatin, downregulates ACSL4 expression, and upregulates SLC7A11 expression. Elevated SENP1 levels are associated with adverse prognoses in lung cancer patients^68^. In cervical cancer (CC) cells, CoCl2-stimulated hypoxia-like conditions specifically enhance the SUMO1 modification of KDM4A at K471, inhibit H3K9me3 levels, upregulate SLC7A11/GPX4, and thereby enhance the resistance of CC cells to ferroptosis^97^. In breast cancer (BC) cells, the protein inhibitor of activated STAT4 (PIAS4) promotes the SUMOylation of SLC7A11 by directly binding to it, while lysine-specific demethylase 1A (KDM1A) acts as its transcriptional activator. Tanshinone IIA reduces the expression of KDM1A, thereby transcriptionally inhibiting the expression of PIAS4. Inhibition of PIAS4-dependent ubiquitination of SLC7A11 further induces ferroptosis, which suppresses the proliferation and metastasis of BC. Therefore, Tanshinone IIA promotes ferroptosis by inhibiting the KDM1A/PIAS4/SLC7A11 axis, thereby inhibiting tumor growth and metastasis^98^.

#### 2.2.2. SUMOylation of Mitochondrial-Related Enzymes

During cisplatin-induced acute kidney injury (AKI), ferroptosis in renal cells is due to the downregulation of DHODH, a key enzyme in mitochondrial pyrimidine synthesis, and its downstream product CoQH2, which exacerbates cisplatin-induced CoQH2 deficiency and LPO, while also causing mitochondrial dysfunction and SIRT3 SUMOylation. Pre-treatment with the mitochondria-targeted antioxidant MitoQ can alleviate cisplatin-induced mitochondrial dysfunction, SIRT3 SUMOylation, and DHODH acetylation, providing a potential therapeutic approach for AKI^99^. Baicalin (BAI) is a bioactive compound with similar cardioprotective effects to the overexpression of SENP1. Sentrin/SENP1 regulates the deSUMOylation process of SIRT3, enhances mitochondrial quality control, prevents cell death, and ultimately improves diabetic cardiomyopathy^100^.

#### 2.2.3. SUMOylation Associated with the Regulation of Immune Responses

Research has elucidated that SUMOylation exerts regulatory influence over ferroptosis and concurrently participates in immune responses. SENP3, identified as a redox-sensitive deubiquitinating protease, assumes a pivotal role in macrophage functionality. SENP3 enhances the susceptibility of macrophages to ferroptosis induced by RSL3, augments ferroptosis in M2 macrophages, and diminishes the proportion of M2 macrophages *in vivo*. SENP3 interacts with and de-SUMOylates FSP1 at the K162 site, thereby heightening macrophage sensitivity to ferroptosis^101^. In melanoma, a novel circular RNA circPIAS1 encodes a unique 108-amino-acid peptide that significantly impedes immunogenic ferroptosis triggered by immune checkpoint blockade (ICB) therapy by regulating the balance between STAT1 SUMOylation and phosphorylation, revealing a new mechanism of immune evasion in melanoma^102^.

#### 2.2.4. SUMOylation Associated with HIF-1α

The novel long non-coding RNA (lnc-HZ06) is capable of concurrently modulating hypoxia (as evidenced by the HIF1α protein), ferroptosis, and miscarriage. Mechanistically, HIF1α-SUMO predominantly functions as a transcription factor to enhance the transcription of NCOA4 in hypoxic trophoblasts. As a transcription factor, HIF1α-SUMO also promotes the transcription of lnc-HZ06, which in turn facilitates the SUMOylation of HIF1α by inhibiting SENP1-mediated de-SUMOylation. Thus, lnc-HZ06 and HIF1α-SUMO form a SUMOylation-related positive feedback regulatory loop^103^. Additionally, HSP70 inhibits ferroptosis through HIF-1α SUMOylation, and the occurrence of lung cancer recurrence subsequent to radiofrequency ablation offers a fresh avenue for exploring therapeutic targets aimed at curbing lung cancer recurrence^101^.

#### 2.2.5. SUMOylation Associated with ACSL4

In the context of spinal cord injury, SENP3 serves as a deubiquitinase for TRIM28. The upregulation of TRIM28, an E3 ubiquitin ligase, leads to the conjugation of SUMO3 to lysine 532 of ACSL4. This modification inhibits the K63 ubiquitination of ACSL4, thereby enhancing its stability and increasing cellular susceptibility to ferroptosis. Additionally, a positive feedback loop between oxidative stress and TRIM28 activity further amplifies ferroptosis. The identification of Rutin hydrate as an inhibitor of the TRIM28/ACSL4 axis presents a novel therapeutic avenue for the treatment of spinal cord injury^104^.

### 2.3. Phosphorylation

**Figure 2:**
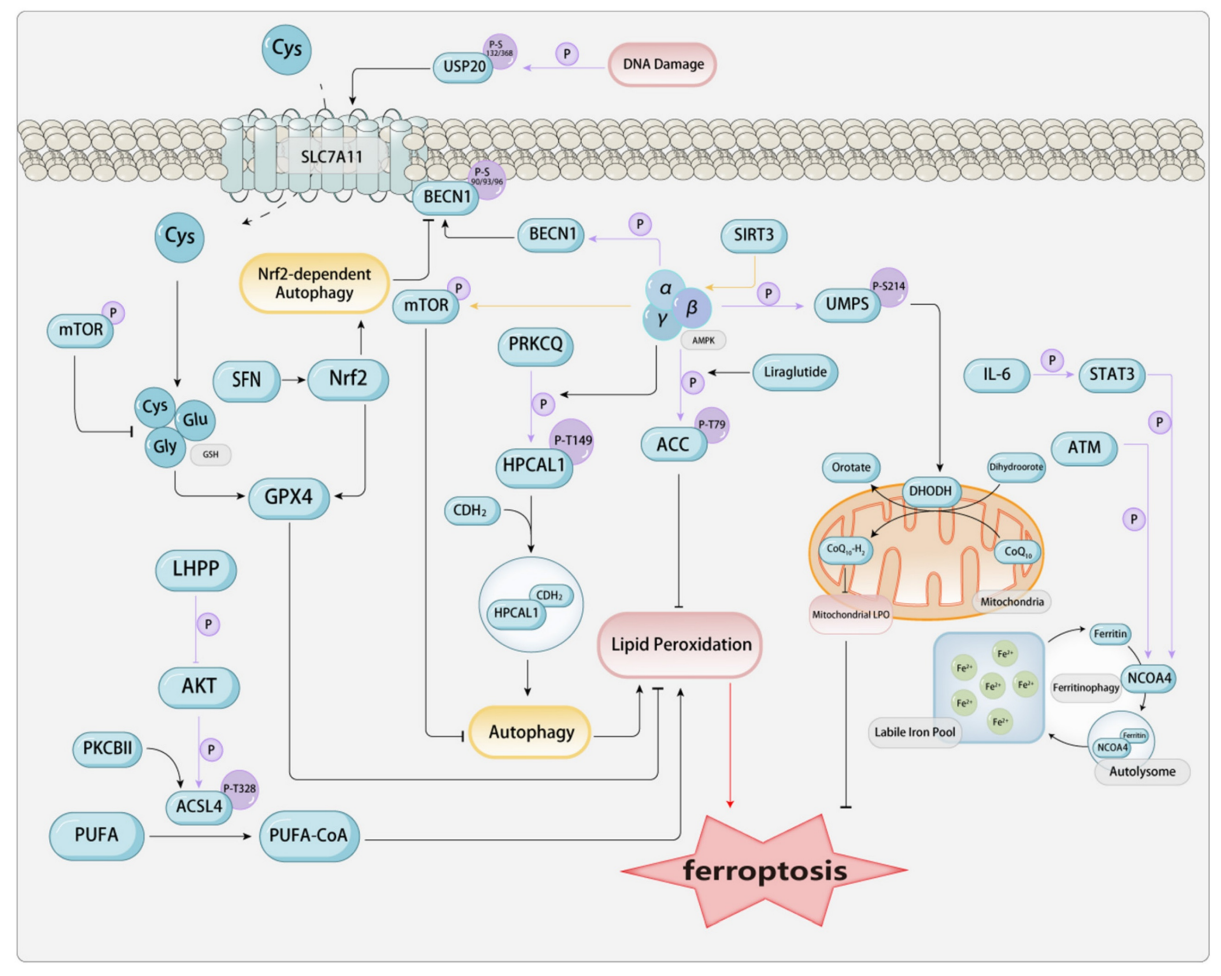
** The phosphorylation in ferroptosis-related proteins. a. Phosphorylation in AMPK-Related Pathways** AMPK is a trimeric complex composed of an α catalytic subunit and β and γ regulatory subunits. Stress conditions such as low ATP levels and hypoxia can activate AMPK, enabling it to phosphorylate downstream proteins for regulatory purposes. ACC is involved in fatty acid oxidation and synthesis. AMPK can phosphorylate the T79 site of ACC, rendering it inactive and thereby reducing lipid deposition. Liraglutide can enhance this process. In contrast, PRKCQ-mediated phosphorylation of HPCAL1 at the Thr149 site can activate the autophagic degradation of CDH2. AMPK-mediated phosphorylation of BECN1 at Ser90/93/96 directly blocks system Xc- by binding to SLC7A11, thereby inhibiting the uptake of Cys and enhancing ferroptosis. SIRT3 positively regulates AMPK; activation of SIRT3 depletes the activation of the AMPK-mTOR pathway, thereby inhibiting autophagy. AMPK phosphorylates UMPS at Ser214, which in turn promotes cell resistance to ferroptosis mediated by DHODH. **b. Ferritin Autophagy-Related Phosphorylation** ATM phosphorylates NCOA4, which dominates the intracellular free iron by promoting the interaction between NCOA4 and ferritin, thereby maintaining ferritinophagy. Meanwhile, the IL-6/STAT3 signaling pathway can upregulate the expression of NCOA4. **c. Phosphorylation Related to ACSL4** Phosphorylation of ACSL4 by PKCβII can promote ferroptosis induced by LPO. In contrast, LHPP interacts with AKT, thereby inhibiting AKT phosphorylation and, consequently, the phosphorylation of ACSL4 at the T624 site. This allows ACSL4 to escape phosphorylation-dependent protein degradation, promoting the accumulation of LPO and ferroptosis. **d. Phosphorylation Related to Nrf2-SLC7A11-GPX4** DNA damage-induced ATR can activate the phosphorylation of USP20 at Ser132 and Ser368, thereby enhancing the stability of SLC7A11. SFN activates the Nrf2 signaling pathway and its downstream targets, enhancing cellular autophagy. Nrf2-dependent autophagy activation disrupts the binding of SLC7A11 to BECN1 phosphorylated at the S93 site and increases the membrane translocation of SLC7A11 to counteract ferroptosis.

Among the many types of post-translational modifications (PTMs), phosphorylation modification is present in approximately one-third of all proteins and ranks as one of the most prevalent and significant types of modifications. Phosphorylation modulates intracellular signaling, cellular architecture, proliferation, apoptosis, transcriptional activity, and metabolic pathways, and the regulatory ability to adapt to pathogenic microorganisms, among other things. Mechanistically, phosphorylation is the process of transferring the γ-phosphate group from ATP or GTP to the side chain of an amino acid in the substrate protein (commonly occurring at serine, threonine, and tyrosine residues), with ATP and GTP being converted to ADP and GDP, respectively. This is a reversible enzymatic reaction, with enzymes that catalyze protein phosphorylation known as protein kinases (PK), and those that catalyze protein dephosphorylation known as protein phosphatases (PPase). Phosphorylation has been found to play a potential role in regulating many metabolic pathways involved in ferroptosis.[**Figure 2**]

#### 2.3.1. Phosphorylation in AMPK-Related Pathways

When it comes to phosphorylation, we must mention one of the classic pathways—the AMPK pathway. Adenosine 5'-monophosphate (AMP)-activated protein kinase (AMPK), also known as AMP-dependent protein kinase, is a central modulator of biological energy metabolism and is indispensable for the maintenance of cellular energy homeostasis. AMPK is composed of a catalytic α subunit and regulatory β and γ subunits, forming a heterotrimeric structure. When AMP binds to the γ subunit, it allosterically activates the complex, making it a more susceptible substrate for phosphorylation at the threonine 172 site, which is more easily phosphorylated in the activation loop of the α subunit by the primary upstream AMPK kinase LKB1. AMPK can also be directly phosphorylated at the threonine 172 site by CAMKK2, a response elicited by fluctuations in intracellular calcium levels subsequent to the activation of metabolic hormones such as adiponectin and leptin. As a kinase, the activity of AMPK is regulated by factors such as the organism's energy status, with an increased AMP/ATP ratio being the main factor that activates AMPK. Generally, stress factors such as tissue ischemia and hypoxia lead to the activation of AMPK. Once activated, AMPK primarily regulates various biological processes by phosphorylating its downstream targets. AMPK, functioning as a cellular energy sensor, detects low ATP levels. Once activated, it enhances signal transduction pathways that restore cellular ATP levels, including fatty acid oxidation and autophagy^15^.

Acetyl-CoA carboxylase (ACC) is the rate-limiting enzyme in fatty acid metabolism, involved in the oxidation and synthesis of fatty acids, and plays a key role in fatty acid metabolism. During stress in the body, AMPK mediates the phosphorylation of ACC, inactivating it by phosphorylating the threonine at position 79, which promotes fatty acid oxidation, reduces serum free fatty acids, and subsequently decreases lipid deposition in tissues, improving lipid metabolism. Investigations have revealed a significant impact of a modest elevation in ACC phosphorylation by AMPK and the phosphorylation of hippocampal calcium-binding protein-like 1 (HPCAL1) at Thr149 mediated by protein kinase C theta (PRKCQ) can activate the autophagic degradation of cadherin 2 (CDH2) and partially protect cells from erastin-induced cell death^103^. Liraglutide is a human glucagon-like peptide-1 (GLP-1) analog produced by recombinant yeast, used for the treatment of adult type 2 diabetes. Liraglutide not only improves glucose metabolism but also regulates lipid metabolism-related signal transduction, including AMPK and ACC. Mechanistically, Liraglutide mitigates type 2 diabetes-associated non-alcoholic fatty liver disease via AMPK/ACC pathway activation and ferroptosis inhibition^105^. Semaglutide (Smg), a GLP-1 receptor agonist, enhances KLB mRNA expression significantly through the activation of the cAMP signaling pathway, particularly via the phosphorylation of PKA and cAMP response element-binding protein (CREB). Subsequently, the AMPK signaling pathway is activated, reprogramming key metabolic processes involved in ferroptosis^106^.

AMPK exerts a significant regulatory influence on the autophagy pathway. AMPK can regulate autophagy-dependent ferroptosis by modulating the core autophagy regulatory factors beclin 1 (BECN1, also known as Atg 6) and the mechanistic target of rapamycin kinase (MTOR). Studies have found that the activation of the BECN1 pathway increases ferroptosis in CRC cells. AMPK-mediated phosphorylation of BECN1 directly blocks system Xc- by binding to SLC7A11, thereby enhancing ferroptosis. Knocking down BECN1 suppresses ferroptosis triggered by system Xc- inhibitors, including erastin, sulfasalazine, and sorafenib. AMPK-induced BECN1 (Ser90/93/96) phosphorylation is a necessary condition for the formation of the BECN1-SLC7A11 complex and LPO. Inhibition of PRKAA/AMPKα can attenuate erastin-induced BECN1 phosphorylation at S93/96, disrupt BECN1-SLC7A11 complex assembly, and inhibit ensuing ferroptosis^62^. SIRT3 positively modulates AMPK phosphorylation. Studies have found that in gestational diabetes, increased expression of SIRT3 leads to classical ferroptosis events and autophagy. Mechanistically, the activation of SIRT3 depletes the activation of the AMPK-mTOR pathway and enhances GPX4 levels, thereby inhibiting autophagy and ferroptosis. Similarly, the depletion of AMPK also blocks the induction of ferroptosis in trophoblasts. Activation of autophagy via SIRT3 upregulation enhances the AMPK-mTOR pathway and reduces GPX4 levels, thereby inducing ferroptosis in trophoblasts^107^. Curcumin (Cur) inhibits autophagy-dependent ferroptosis via the Sirt1/AKT/FoxO3a signaling pathway, thereby maintaining cardiomyocyte function^108^. Piezo1 is a member of the mechanosensitive cation channel family. Studies have found that mice with specific intestinal epithelial Piezo1 deficiency (Piezo1ΔIEC) show significantly diminished intestinal inflammation and enhanced intestinal barrier function relative to wild-type (WT) mice in dextran sulfate sodium (DSS)-induced colitis. Erastin is capable of reversing the protective impact of Piezo1 silencing on LPS-induced ferroptosis in Caco-2 cells. Mechanistically, Piezo1 has been found to modulate ferroptosis via the AMPK/mTOR signaling pathway^109^.

Additionally, AMPK can also affect the phosphorylation of metabolic pathways. DHODH reduces CoQ's involvement in pyrimidine metabolism by promoting the oxidation of dihydroorotate (DHO) into orotate. AMPK phosphorylates the Ser 214 site of orotate monophosphate synthase (UMPS) and enhances pyrimidine body assembly, thereby promoting DHODH-mediated resistance to ferroptosis in HeLa cells^110^.

Since AMPK is closely related to energy metabolism, and mitochondria are the most important energy factories in cells, they play a significant role in regulating ferroptosis. AMPK is also crucial for regulating mitochondrial homeostasis. Therefore, researchers have compared the main differences between mitochondrial and AMPK functions in ferroptosis. Although mitochondria selectively promote ferroptosis induced by cysteine starvation or erastin, but not by RSL3. However, studies have shown that glucose deprivation or AMPK deficiency sensitizes cells to ferroptosis induced by erastin, RSL3, and cysteine starvation. Thus, energy stress-mediated AMPK activation may inhibit ferroptosis through mitochondrial-independent mechanisms^111^. Nicorandil treatment enhances the phosphorylation of AMPKα1 and promotes its translocation to the mitochondria, thereby inhibiting the mitochondrial translocation of ACSL4, which is typically mediated by mitophagy. Consequently, this process suppresses mitochondria-associated ferroptosis^112^.

Due to the multitude of downstream signals of AMPK, this provides more possibilities for targeting the phosphorylation regulation of ferroptosis through AMPK-related pathways. However, related research is still somewhat lacking, and we need more studies on its regulation of energy metabolism, lipid metabolism, and autophagy-related pathways under stress and non-stress conditions.

##### 2.3.2. Ferritin Autophagy-Related Phosphorylation

Ferritin autophagy, a critical component of ferroptosis, is primarily regulated by NCOA4. Mechanistically, NCOA4-mediated ferritin autophagy plays a crucial role in maintaining intracellular iron homeostasis by promoting ferritin transport and iron release^113^. The Ser/Thr kinase ATM serves as the principal detector of DNA double-strand break damage. Studies have reported that ATM phosphorylates NCOA4 to dominate the intracellular unstable free iron, promoting the interaction between NCOA4 and ferritin, thereby maintaining ferritin autophagy^114^. Additionally, the phosphorylation of pathway proteins related to NCOA4 also has a significant impact on ferroptosis. Studies have found that the IL-6/STAT3 signaling pathway modulates NCOA4 expression. Inhibition or knockdown of STAT3 can effectively reduce NCOA4 levels, protecting H9C2 cells from ferroptosis mediated by ferritin autophagy, while overexpression of STAT3 via plasmids appears to augment NCOA4 expression, culminating in canonical ferroptosis events. The upregulation of phosphorylated STAT3, activation of ferritin autophagy, and induction of ferroptosis also occur in mice fed a high-fat diet (HFD), and are the cause of HFD-induced cardiac damage. Evidence suggests that Piperlongumine, a natural compound, can effectively reduce the levels of phosphorylated STAT3 both *in vitro* and *in vivo*, protecting cardiomyocytes from iron autophagy-mediated ferroptosis^115^. Further understanding of NCOA4-mediated ferritin autophagy, as well as the modifications of the NCOA4 protein itself, may help in the development of new therapeutic strategies for diseases involving ferritin autophagy.

#### 2.3.3. Phosphorylation Related to ACSL4

ACSL4 is an important enzyme that induces the occurrence of ferroptosis in the lipid metabolic pathway. The biosynthesis of arachidonyl-CoA catalyzed by ACSL4 contributes to the execution of ferroptosis by triggering phospholipid peroxidation^116^. Studies have found that the phosphorylation of ACSL4 by PKCβII promotes ferroptosis induced by LPO. PKCβII detects initial lipid peroxides and amplifies ferroptosis-associated LPO via ACSL4 phosphorylation and activation, emerging as a crucial ferroptosis mediator. Suppressing the PKCβII-ACSL4 pathway mitigates ferroptosis *in vitro* and obstructs immunotherapy-induced ferroptosis *in vivo*^16^. LHPP, a tumor suppressor, modulates diverse signaling pathways via its phosphatase activity, having a key impact on the regulation of tumor cell proliferation and survival. Studies have shown that LHPP interacts with Protein Kinase B (also known as AKT), thereby inhibiting AKT phosphorylation, which in turn suppresses the phosphorylation of ACSL4 at the T624 site. This interaction obstructs phosphorylation-dependent ubiquitination, thereby inhibiting SKP2 from recognizing and binding to ACSL4 at the K621 site. Consequently, ACSL4 escapes lysosomal degradation, resulting in its accumulation and the promotion of LPO and ferroptosis^117^. Protosappanin A (PrA) is an active compound extracted from the traditional Chinese medicinal herb, Sappan wood. Recent studies have demonstrated that PrA binds to ACSL4 and FTH1, thereby inhibiting ACSL4 phosphorylation and phospholipid peroxidation, while preventing the autophagic degradation of FTH1 and the release of Fe²⁺^118^.

#### 2.3.4. Phosphorylation Related to Nrf2-SLC7A11-GPX4

GPX4 is an important catalytic enzyme in the antioxidant mechanism against ferroptosis. Phosphorylation modifications of GPX4, as well as modifications of system Xc-, which significantly affect the function of GPX4, can regulate ferroptosis. Research has demonstrated that the activation of the insulin-like growth factor 1 receptor (IGF1R) signal activates AKT to phosphorylate CKB at the T133 site, diminishing its metabolic activity while augmenting its interaction and phosphorylation with GPX4 at the S104 site. This mechanism blocks the interaction between HSC70 and GPX4, thereby preventing GPX4 degradation via chaperone-mediated autophagy in mice, inhibiting ferroptosis, and promoting tumor growth^12^. Additionally, investigations have revealed that USP20 is highly expressed in HCC cells exhibiting resistance to OXA. Elevated USP20 levels in HCC correlate with adverse prognostic outcomes. USP20 facilitates OXA resistance and acts as a suppressor of ferroptosis in HCC. Immunoprecipitation results show that the UCH domain of USP20 engages in an interaction with the N-terminus of SLC7A11. USP20 enhances the stability of SLC7A11 by deubiquitinating the K48-linked polyubiquitin chains at the K30 and K37 residues of the SLC7A11 protein. Most importantly, the phosphorylation of USP20 at Ser132 and Ser368 requires the activation of DNA damage-induced ATR. The phosphorylation of USP20 at Ser132 and Ser368 augments its stability, thereby conferring OXA and ferroptosis resistance to HCC cells^119^. Furthermore, certain herbal extracts have been shown to exert therapeutic effects. Bergapten, a natural coumarin derivative found in citrus peel, has been reported to inhibit PI3K phosphorylation and indirectly restore GPX4 expression, thereby suppressing ferroptosis and alleviating renal fibrosis^120^.

Nrf2 serves as a pivotal modulator of cellular antioxidant defenses and an upstream gene of SLC7A11 and GPX4. It mitigates ferroptosis through the induction of autophagy and regulation of genes associated with iron homeostasis and GSH biosynthesis/metabolism. Studies have found that sulforaphane (SFN) intervention can modulate Nrf2 and autophagy pathways to inhibit ferroptosis in acute liver injury (AKI). The findings indicate that SFN activates the Nrf2 signaling pathway and its downstream targets, enhances cellular autophagy, and thereby counteracts ferroptosis to mitigate liver injury. Upon Nrf2 inhibition, SFN-induced autophagy nearly ceases, and the anti-ferroptosis efficacy is markedly diminished. When autophagy is inhibited, SFN retains the ability to activate Nrf2 and its downstream targets, yet the membrane translocation of SLC7A11 and its cystine transport function are substantially diminished, ultimately attenuating SFN's anti-ferroptosis effect. Subsequent investigations have revealed that Nrf2-dependent autophagy activation disrupts the binding of SLC7A11 with BECN1 phosphorylated at S93 and augments the membrane translocation of SLC7A11 to counteract ferroptosis. In summary, the activation of autophagy dependent on Nrf2 is vital for facilitating SLC7A11 membrane localization to counteract ferroptosis. The activation of Nrf2 not only upregulates the expression of SLC7A11, GPX4, and autophagy-related proteins but also promotes SLC7A11 membrane transfer and GSH synthesis by inducing autophagy to disrupt the binding of SLC7A11 and BECN1, ultimately inhibiting ferroptosis^121^. Modulating Nrf2 phosphorylation is a potential chemotherapy target. Delavinone inhibits PKCδ kinase activity, reducing Nrf2 nuclear translocation and GSH-related gene expression, thereby inducing ferroptosis in CRC cells. However, GPX4 overexpression attenuates delavinone's anticancer effects^122^. Consuming fermented foods and beverages exposes individuals to ethyl carbamate (EC). EC, known as an environmental toxin, can cause severe toxicity mediated by oxidative stress. Studies have found that ferroptosis may be one of the pathways through which EC exerts its toxicity. EC-triggered GSH depletion mainly depends on the inhibition of GSH synthesis by suppressing the expression of SLC7A11 and GCLC. Notably, EC blocks the activation of Nrf2 by inhibiting phosphorylation modifications and nuclear translocation, which further leads to the occurrence of ferroptosis^123^. Inhibition of APE1 enhances ferroptosis by modulating the NRF2/SLC7A11/GPX4 axis, which regulates redox activity. Genetic or chemical inhibition of APE1 increases AKT oxidation, impairs AKT phosphorylation, and dephosphorylates and activates GSK3β, ultimately promoting NRF2 ubiquitin-proteasome-dependent degradation^124^. In summary, phosphorylation of relevant proteins that regulate NRF2 can control the expression of SLC7A11 and GPX4, thereby triggering ferroptosis and providing potential therapeutic strategies for ferroptosis-based disease treatment.

### 2.4. Acetylation

**Figure 3:**
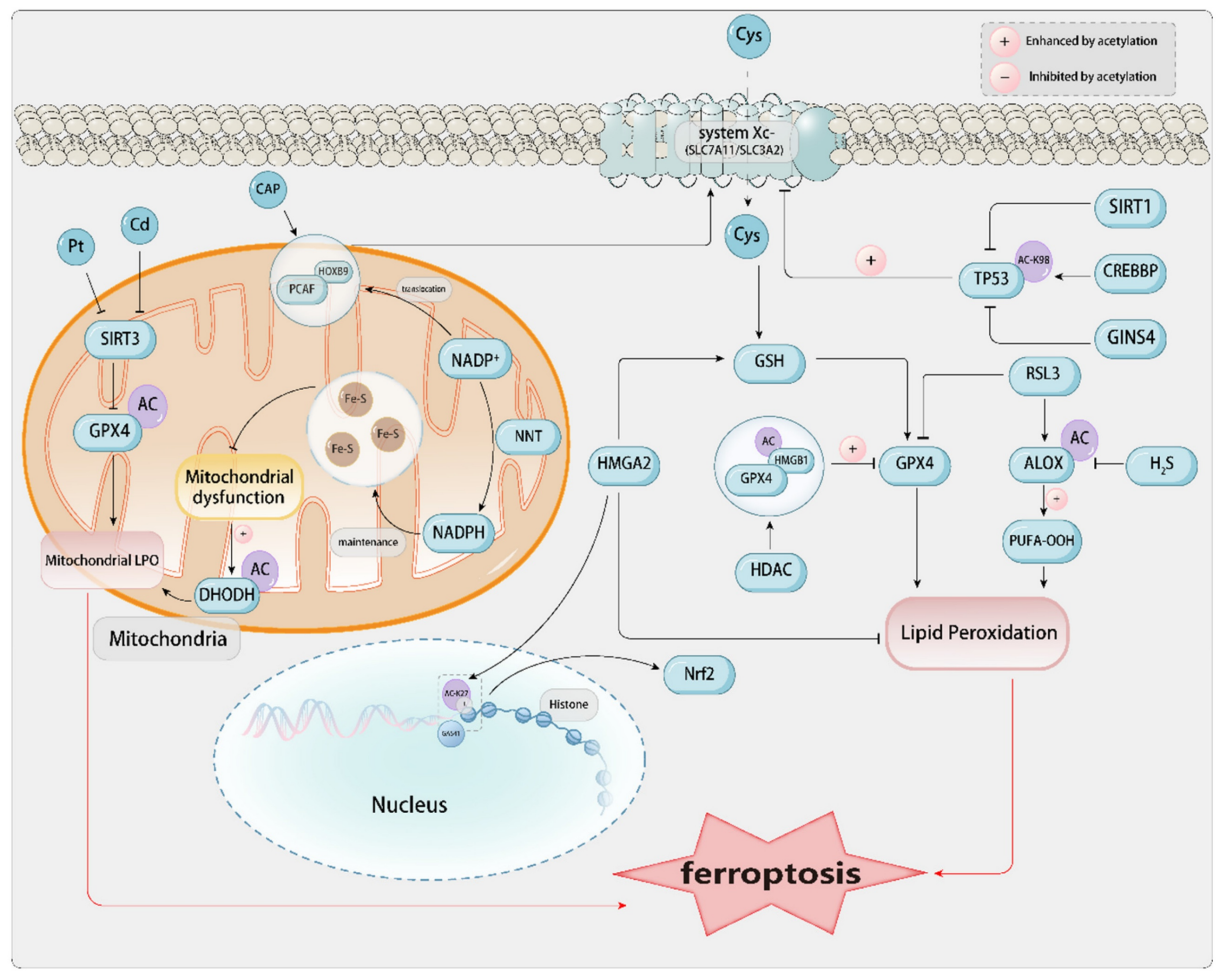
** The acetylation in ferroptosis-related proteins.** Intracellular Cys is imported and synthesized into GSH via system Xc- (SLC7A11/SLC3A2). CREBBP can promote the acetylation of TP53 at K98, which inhibits the expression of SLC7A11 and reduces Cys uptake. Conversely, SIRT1 can deacetylate TP53, thereby increasing Cys uptake and inhibiting the occurrence of ferroptosis. Within the mitochondria, SIRT3 deacetylates mitochondrial GPX4, thereby alleviating the lipid peroxidation of the mitochondrial membrane. CAP promotes the interaction between HOXB9 and PCAF, increasing the acetylation level of HOXB9, which in turn affects its ubiquitination status and influences HOXB9-regulated ferroptosis mediated by SLC7A11. Under IL-1β stimulation, cancer cells exhibit NNT, which triggers the mitochondrial translocation of PCAF. Acetylation enhances the binding affinity for NADP+, thereby augmenting NNT activity, which in turn promotes the synthesis of NADPH to maintain iron-sulfur clusters and suppress mitochondrial dysfunction. Conversely, mitochondrial dysfunction induces the acetylation of DHODH, partially exacerbating ferroptosis, worsening cisplatin-induced CoQH2 deficiency, and LPO. In the regulation of gene expression, HMGA2 activates GPX4 expression at the transcriptional and translational levels by increasing the methylation of H3K4 and the acetylation of H3K27, thereby inhibiting the production of ROS and promoting the GSH. Similarly, GAS41 can recognize H3K27-acetylation and enhance the transcriptional capacity of NRF2 on antioxidant genes. HDAC can mediate the shuttling of HMGB1 between the nucleus and the cytoplasm. Acetylated HMGB1 can interact with GPX4, thereby inhibiting the ferroptosis-resistant activity of GPX4. RSL3 promotes the protein expression and acetylation of ALOX12. In contrast, the introduction of NaHS reduces the acetylation of ALOX12, thereby protecting against membrane LPO.

Recent studies indicate that the acetylation of various proteins, such as tumor protein p53 (TP53), ALOX12, and HMGB1, is implicated in the modulation of ferroptosis. TP53 predominantly functions as a transcription factor and a potent tumor suppressor through apoptosis induction, and it assumes a distinctive role in the modulation of ferroptosis upon alterations in its acetylation. In H1299 lung cancer cells, the acetylation-deficient mutant TP53^3KR^ triggers ferroptosis by downregulating SLC7A11. CREB-binding protein (CREBBP, also known as CBP), serving as a pivotal acetyltransferase for TP53 acetylation, downregulates the transcriptional expression of SLC7A11 in a model of acute lung injury. In contrast, TP53 deacetylation mediated by sirtuin 1 (SIRT1) inhibits heat stress-induced ferroptosis in lung epithelial cells. The acetylation exquisitely modulates the activity of ALOX12, thereby augmenting its pivotal role in the regulation of ferroptosis. In comparison, hydrogen sulfide (H_2_S) synthesized by cystathionine γ-lyase (CTH, also known as CSE) inhibits the acetylation of ALOX12 and protects myoblasts from ferroptosis. Additionally, acetylated HMGB1 can interact with GPX4, inhibiting GPX4's anti-ferroptotic activity in colorectal cancer cells^9^.[**Figure 3**]

#### 2.4.1. Acetylation Related to P53

p53, especially the acetylation-deficient mutant p53, functions as a positive regulator of ferroptosis by suppressing the expression of SLC7A11^125^. p53 inhibits the uptake of cystine and sensitizes cells to ferroptosis by suppressing the expression of SLC7A11. The p533KR mutant, incapable of inducing cell cycle arrest, senescence, or apoptosis, maintains the capacity to modulate SLC7A11 expression and trigger ferroptosis under ROS-induced stress. High expression of SLC7A11 in human tumors can inhibit ROS-induced ferroptosis and eliminate p53^3KR^-mediated tumor growth inhibition in xenograft models^126^. Researchers have identified the acetylation site lysine K98 in mouse p53 (or human p53's K101). When all four acetylation sites are mutated simultaneously (p53^3KR^: K98R+3KR(K117R+K161R+K162R)), It entirely nullifies its capacity to modulate metabolic targets (such as TIGAR and SLC7A11), severely impairing the transcriptional activity of p53. Notably, the acetylation of K98 in mouse p53 is crucial for suppressing the expression of the SLC7A11 gene and p53-mediated ferroptosis, and the lack of K98 acetylation impairs the tumor suppressor function of p53. In the absence of other key acetylation sites in the DNA-binding domain, the absence of K98 acetylation will prevent p53 from mediating ferroptosis through acetylated targets and effectively inhibiting tumor growth^127^. Additionally, research data indicate that GINS complex subunit 4 (GINS4), as a promoter of eukaryotic G1/cell cycle, is considered a potential oncogene in LUAD. It destabilizes p53 through the inhibition of p53 acetylation, thereby inhibiting ferroptosis and presenting a potential therapeutic target for LUAD^128^.

Calcium oxalate (CaOx) calculi represent one of the predominant forms of renal lithiasis. Research results indicate that the expression of p53 increases in patients with kidney stones and in mice with high blood oxygen, and ferroptosis is significantly activated under oxalate stimulation by enhancing the acetylation of p53. The activation of the deacetylase SIRT1 or the triple mutation of p53 induces the deacetylation of p53, inhibits ferroptosis, and alleviates renal fibrosis caused by CaOx crystals. The modulation of ferroptosis via SIRT1-mediated deacetylation of p53 may serve as a potential therapeutic target for preventing renal fibrosis in individuals with kidney stones^129^. Typical characteristics of ferroptosis can also be exhibited during the death of cardiomyocytes during heart failure. SIRT proteins may serve as potential therapeutic targets for cardiac fibrosis and heart failure. Downregulating the level of GPX4 can mediate the knockout of SIRT3, which in turn can promote ferroptosis and cardiac fibrosis through p53 acetylation. In addition, circular RNA FEACR (ferroptosis-related circular RNA) regulates ferroptosis in cardiomyocytes. It directly interacts with nicotinamide phosphoribosyltransferase (NAMPT), thereby maintaining its stability, which in turn influences the NAD-dependent deacetylation of forkhead box protein O1 (FoxO1) by SIRT1, and subsequently modulates the transcription of ferritin heavy chain 1 (Fth1), thereby inhibiting ferroptosis in cardiomyocytes^130^. The regulation of ferroptosis in cardiomyocytes involving the circular RNA FEACR-mediated NAMPT-Sirt1-FOXO1-FTH1 signaling axis can also protect cardiac function from I/R injury^131^. Irisin, a cleavage peptide highly expressed and released in cardiomyocytes, exhibits therapeutic efficacy in cardiovascular diseases, including cardiac ischemia/reperfusion (I/R), atherosclerosis, cardiac hypertrophy, and injury. It ameliorates cardiac function in type 1 diabetes by inhibiting ferroptosis via the SIRT1-p53-SLC7A11/GPX4 pathway. Under the action of irisin, the upregulation of SIRT1 levels reduces the acetylation of p53 K382, thereby downregulating the expression level of p53 protein and upregulating the expression of SLC7A11 and GPX4. This protects cardiomyocytes from injury caused by high glucose^132^. Some studies have also demonstrated that in the cardiovascular disease aortic dissection (AD), the downregulation of P300 levels promotes the binding of p53 to HIF-1α, thereby upregulating the expression level of HMOX1 and exacerbating ferroptosis^133^. In addition, studies on traditional Chinese medicine have shown that paeoniflorin, an active component of peony, can significantly improve neuronal damage after brain injury by inhibiting mitochondrial damage, increasing GPX4 activity, reducing MDA production, restoring nerve function, and inhibiting brain edema. Paeoniflorin promotes p53 ubiquitination and degradation via the proteasome, inhibits p53 acetylation, reduces its stability, and regulates the SLC7A11-GPX4 pathway to inhibit ferroptosis^134^.

#### 2.4.2. Acetylation Related to ALOX12

ALOX12, a member of the lipoxygenase (ALOX) family, encodes arachidonic acid 12-lipoxygenase. This enzyme catalyzes the conversion of polyunsaturated fatty acid substrates into bioactive lipid mediators. ALOX12 holds a significant research position in cancer and is closely associated with processes such as cell proliferation, apoptosis, and ferroptosis. Studies have found that H_2_S derived from CSE has been proven to regulate oxidative stress and lipid metabolism. The ferroptosis agonist RSL3 inhibits the expression of GPX4, reduces CSE/H_2_S signaling, resulting in elevated oxidative stress and LPO, and ferroptosis. Exogenous application of NaHS can also block RSL3-induced ferroptosis. Moreover, H2S mitigates RSL3-induced Drp1 upregulation and mitochondrial impairment, thereby ameliorating aberrant lipid metabolism. RSL3 promotes the protein expression and acetylation of ALOX12, a pivotal protein initiating membrane phospholipid oxidation, whereas the introduction of NaHS diminishes ALOX12 acetylation, protecting against membrane LPO. The findings demonstrate that the suppression of the CSE/H2S signaling pathway results in mitochondrial dysfunction, aberrant lipid metabolism, membrane LPO, and ferroptosis. Targeting the CSE/H2S system may serve as a strategy to inhibit ferroptosis in skeletal muscle^135^.

#### 2.4.3. Acetylation Related to HMGB1

HMGB1, a non-histone nuclear protein, has its localization and activity modulated by post-translational modifications. HMGB1 shuttles between the nucleus and the cytoplasm through acetylation and deacetylation modifications of NLS1 and NLS2, mediated by proteins of the histone acetyltransferase (HAT) family and the histone deacetylase (HDAC) family. Conversely, acetylation of lysine residues within the NLS can inhibit the nuclear re-entry of HMGB1. Additionally, HMGB1 can bind to exportin 1, undergo acetylation under oxidative stress, and subsequently translocate to the cytoplasm. Animal studies have confirmed that various oxidative stresses can induce the release of highly acetylated HMGB1 under various pathological conditions The translocation of Cathepsin B from the lysosome to the nucleus induces DNA damage, leading to the subsequent release of HMGB1, which can induce the occurrence of ferroptosis^136^.

#### 2.4.4. Acetylation Related to Mitochondria

Mitochondrial SIRT3, an NAD^+^-dependent deacetylase that modulates the stability of mitochondrial proteins, is diminished in the kidneys of mice exposed to cadmium, coinciding with reduced mitochondrial cristae levels, heightened GPX4 acetylation, and decreased mitochondrial GPX4 protein. In SIRT3 knockout mice, cadmium-induced mitochondrial GPX4 acetylation and renal cell ferroptosis are exacerbated. The findings indicate that the downregulation of SIRT3 may result in mitochondrial GPX4 acetylation, which is implicated in cadmium-induced ferroptosis in renal cells^137^. During cisplatin-induced AKI, mitochondrial dysfunction-induced DHODH acetylation partially promotes renal cell ferroptosis. The key enzyme for mitochondrial synthesis of pyrimidines, DHODH, and its downstream product CoQH_2_ are downregulated. The silenced expression of DHODH will exacerbate cisplatin-induced CoQH_2_ deficiency and LPO. An increase in DHODH acetylation is observed in the kidneys of mice exposed to cisplatin. The kidneys and HK-2 cells treated with cisplatin show a downregulation of mitochondrial SIRT3^99^.

#### 2.4.5. Acetylation and Cancer

As a PTM, acetylation has attracted considerable attention in various cancers. Studies have delved into the potential impact of acetylation on the pathological mechanisms and therapeutic strategies of cancer. In colorectal cancer tissues and various colon cancer cell lines, the expression of N-acetyltransferase 10 (NAT10) is upregulated. The upregulated expression of NAT10 in colon cancer cells is associated with a shorter survival period. The inhibition of ferroptosis reverses the inhibition of colon cancer cell proliferation and metastasis caused by the knockdown of NAT10. NAT10 can also improve the stability of FSP1 mRNA, enhancing the expression of FSP1 in colon cancer cells. The N4 acetylation modification of FSP1 mRNA is associated with the inhibition of ferroptosis, and the knockdown of NAT10 significantly increases ferroptosis in colon cancer cells^138^. Recent studies have demonstrated that the preservation of FSP1 acetylation is intricately linked to SLC25A1, thereby effectively preventing its proteasomal degradation. SLC25A1 facilitates the transport of citrate from the mitochondria to the cytoplasm, where it is subsequently converted to acetyl-CoA by ATP citrate lyase (ACLY) to sustain FSP1 acetylation. Notably, the inhibition of both SLC25A1 and ACLY markedly increases the susceptibility of cancer cells to ferroptosis, thus presenting a novel therapeutic target for cancer therapy^139^. High mobility group AT-hook 2 (HMGA2) is highly expressed in pancreatic cancer tissues. Cells overexpressing HMGA2 significantly inhibit ferroptosis by suppressing ROS and promoting the production of GSH. On the contrary, the absence of HMGA2 exacerbates ferroptosis. In the activation of GPX4 expression regulated by HMGA2 transcription and translation, the methylation of H3K4 and the acetylation of H3K27 are increased^140^. The acetylation of H3K27, upregulated by HPV oncogenes E6 and E7 via CBP/p300, increases the expression of ESCO1 and lncTUBA3FP. TUBORF, encoded by lncTUBA3FP, is highly expressed in CC. TUBORF's binding and acetylation of ESCO1 degrade immune-related GTPase Q, inhibiting ferroptosis and promoting cancer cell proliferation. However, silencing ESCO1 or TUBORF enhances paclitaxel's anticancer efficacy, offering new insights for CC treatment^141^. Ovarian cancer (OC) is mainly treated with platinum-based chemotherapy; however, platinum resistance (Pt-R) has emerged as a pressing clinical challenge. Recent studies have demonstrated that HDL-like nanoparticles (HDL NPs) with low cholesterol content, when used to block scavenger receptor B-1 (SR-B1), can reduce cholesterol uptake and decrease the acetylation of lysine 27 on histone H3, thereby inducing ferroptosis and inhibiting tumor growth both *in vitro* and *in vivo*^142^.

GAS41 protein is a histone reader and an oncogene, which is recruited to the SLC7A11 promoter by recognizing the H3K27-acetylated (H3K27-ac) mark, interacts with NRF2, and promotes NRF2's transcriptional ability on antioxidant genes, thereby inhibiting ferroptosis^143^. Cold atmospheric plasma (CAP) technology is promising due to its ability to induce oxidative stress in malignant tissues without causing thermal damage. CAP can effectively suppress the proliferation and metastatic potential of pulmonary carcinoma cells and upregulate ferroptosis. CAP promotes the interaction between HOXB9 and the acetyltransferase p300/CBP-associated factor (PCAF), increasing the acetylation level of HOXB9. HOXB9 acetylation influences its ubiquitination status, consequently impacting its protein stability. Low expression of HOXB9 reduces the expression of SLC7A11, and CAP regulates ferroptosis mediated by SLC7A11 through the modulation of HOXB9^144^. Interleukin-1β (IL-1β) is an essential mediator of inflammation and is implicated in tumor progression. Upon IL-1β stimulation, cancer cells exhibit acetylation of nicotinamide nucleotide transhydrogenase (NNT) at lysine 1042 (NNT K1042ac), which triggers the mitochondrial translocation of p300/CBP-associated factor (PCAF). The acetylation augments NNT activity through heightened NADP^+^ binding affinity, consequently boosting NADPH synthesis to sustain iron-sulfur clusters and shield tumor cells against ferroptosis^145^. The role of ferroptosis in cancers induced by oncogenic Kaposi's sarcoma-associated herpesvirus (KSHV) remains elusive. The deacetylation of SERPINE1 mRNA-binding protein 1 (SERBP1) is required for KSHV-induced cell transformation. Mechanistically, the viral interleukin-6 (vIL-6) encoded by KSHV facilitates SIRT3-mediated deacetylation of SERBP1, preventing its binding to lipoyl transferase 2 (Lipt2) mRNA and protecting it from mRNA degradation-induced ferroptosis^146^.

Existing research has revealed that the upregulation of enzymes and genes associated with acetylation can inhibit ferroptosis in cancer cells. Nevertheless, the mechanisms of different acetylation-related enzymes and genes and the specificity of acetylation in different cancers still require more in-depth and extensive investigation.

### 2.5. Methylation

Protein and nucleic acid methylation, a key epigenetic mechanism, modulates genetic activity and quiescence, and is implicated in the etiology of diverse disorders, including oncogenesis, senescence, and neurodegenerative conditions like Alzheimer's. The most common methylation modifications are DNA methylation and histone methylation. Mechanistically, methylation refers to the process of catalytically transferring a methyl group from an active methyl compound to other compounds, forming various methylated compounds or chemically modifying certain proteins or nucleic acids to form methylated products. In recent years, an increasing number of epigenetic studies have proven that methylation of DNA, RNA, and proteins plays a significant role in ferroptosis^147^.

#### 2.5.1. Histone Methylation

Histone methylation is one of the most common types of protein methylation. It primarily refers to the methylation of lysine and arginine residues at the N-terminus of histone tails, which are crucial for the establishment and maintenance of heterochromatin structure, genomic imprinting, DNA repair, X-chromosome inactivation, and transcriptional regulation. Histone methylation necessitates the participation of histone methyltransferases (HMTs), which encompass histone lysine methyltransferases (HKMTs) and protein arginine methyltransferases (PRMTs). Lysines on histones can be mono-methylated, di-methylated, or tri-methylated (denoted as me1, me2, or me3, respectively). Although there is limited research on the role of histone methylation in ferroptosis, it is possible to demonstrate that histone methylation plays a significant role in ferroptosis within cells. PRMTs exert direct and indirect control over the activity and expression of GPX4 via methylation, thus emerging as key regulators of ferroptosis. This regulatory pathway encompasses multiple molecular levels, including DNA, RNA, and protein. However, the hierarchical interplay among these modifications and the potential presence of negative feedback or bidirectional regulation are yet to be elucidated, necessitating further research^148^.

Studies have shown that HKMTs may promote ferroptosis. Lysine-specific methyltransferase 2B (KMT2B), which belongs to the histone H3 lysine 4 (H3K4) methyltransferase family, accelerates the transcription of riboflavin kinase (RFK) by enhancing H3 methylation levels, thereby activating the TNF-α/NOX2 pathway and promoting ferroptosis induced by myocardial ischemia-reperfusion^149^. Similarly, PRMTs also appear to have the effect of promoting ferroptosis. The downregulation of protein arginine methyltransferase 7 (PRMT7) can inhibit ferroptosis in pulmonary epithelial cells caused by inflammatory monocytes, while PRMT7 promotes this process, thereby facilitating the adhesion and transmigratory migration of monocytes in patients with chronic obstructive pulmonary disease (COPD)^150^.

H3K27 refers to the lysine residue at the 27th position of histone H3 (Lysine 27), which can also undergo methylation modification. H3K27 methylation is a pivotal histone mark for the repression of gene expression, primarily catalyzed by the enzyme enhancer of zeste homolog 2 (EZH2)^151^. The hepatitis B virus X protein (HBx) suppresses the expression of SLC7A11 through EZH2-mediated H3K27me3 modification, thereby promoting D-GalN-induced hepatotoxicity and ferroptosis^152^. However, EZH2 alleviates ferroptosis in tongue squamous cell carcinoma cells by upregulating the expression of SLC7A11 through the suppression of miR-125b-5p^153^. One of the salient features of AD is the loss of VSMCs. Studies have indicated that ferroptosis is one of the causes of this loss. SP2509, as a Lysine-Specific Demethylase 1 (LSD1) inhibitor, dose-dependently upregulates the dimethylation of lysine residue at position 4 of histone H3 (H3K4me2), which is downregulated by ferroptosis, through the inhibition of LSD1. This in turn suppresses the efficiency of iron ion uptake by TFR and the iron storage capacity of ferritin, leading to a decrease in intracellular iron ion levels and inhibition of ferroptosis. By inhibiting ferroptosis in VSMCs, SP2509 reduces the degree of aortic structural and functional damage and lowers the risk of AD^154^. Another study has found that the histone methyltransferase inhibitor BRD4770 activates the classical antioxidant pathway of ferroptosis by inhibiting the mono-methylated, di-methylated, or tri-methylated of lysine residue at position 9 of histone H3 (H3K9me1/2/3). This leads to the downregulation of TFR, HMOX1, ferritin, and 4-hydroxynonenal (4-HNE) levels, thereby inhibiting ferroptosis. BRD4770 can also effectively protect VSMCs and slow the progression of AD^155^. Ferroptosis occurs during the progression of AD, and its inhibition can in turn affect the development of AD by protecting VSMCs and alleviating the pathological process of AD. The inhibition of ferroptosis can be achieved by targeting the methylation modifications of different histone sites. Interestingly, SP2509 inhibits ferroptosis by activating H3K4me2, while BRD4770 inhibits ferroptosis by silencing H3K9me1/2/3. Both compounds can protect VSMCs and mitigate the destruction of aortic structure and function, indicating that targeting histone methylation and the ferroptosis pathway holds promise for the treatment of AD.

#### 2.5.2. Non-histone Methylation

Non-histone methylation is another type of methylation modification, which primarily refers to the methylation of the ε-amino group of lysine and the guanidino group of arginine in non-histone proteins. Lysine can be monomethylated, dimethylated, or trimethylated, while arginine can be monomethylated or dimethylated. Ferroptosis in AD can target not only histone methylation but also non-histone methylation. In the aortas of AD patients, the expression of the m6A methyltransferase METTL3 is significantly upregulated. The m^6^A modification of SLC7A11 and FSP1 mRNA by METTL3 promotes the degradation of these mRNAs, thereby downregulating the levels of SLC7A11 and FSP1 and promoting ferroptosis. However, the knockdown of METTL3 can reverse this effect and influence the occurrence and development of AD^156^. In glioblastoma multiforme (GBM), METTL3 interacts with the C5aR1-activated Extracellular Signal-Regulated Kinase 1/2 (ERK1/2) signaling pathway by directly binding to phosphorylated ERK1/2. This interaction enhances the stability of METTL3 protein and stabilizes its m6A methylation modification of GPX4 mRNA, thereby upregulating the expression of GPX4 and inhibiting ferroptosis. This process subsequently promotes the pathological progression of GBM. However, the knockdown of C5aR1 or the use of the C5aR1 inhibitor PMX205 can reverse this effect, indicating potential therapeutic prospects for the treatment of GBM^157^. Protein arginine methyltransferases (PRMTs) primarily influence protein function through the methylation of arginine residues. They can affect ferroptosis both by histone methylation and by non-histone methylation. For example, PRMT6 catalyzes the asymmetric dimethylation of the arginine residue at position 2 of histone H3 (H3R2me2a), leading to the downregulation of GPX4 expression and the promotion of ferroptosis. PRMT8 catalyzes the monomethylation or asymmetric dimethylation of the arginine residue at position 3 of histone H2 or H3 (H4R3me1, H4R3me2a, H2AR3me1, H2AR3me2a), resulting in the upregulation of GPX4 expression and the inhibition of ferroptosis. PRMTs can also affect ferroptosis through the methylation of non-histone proteins. For instance, PRMT4 methylates the arginine residue at position 339 of ACSL4, leading to an increase in reactive oxygen species (ROS) levels and the promotion of ferroptosis. PRMT5 symmetrically dimethylates the arginine residue at position 549 of ACSL4, inhibiting lipid peroxidation and thus suppressing ferroptosis. Additionally, PRMT5 can symmetrically dimethylate the arginine residue at position 152 of GPX4, enhancing GPX4 stability and inhibiting ferroptosis^148^,^158^; PRMT7 methylates mitochondrial ribosomal protein S23, leading to mitochondrial dysfunction and the promotion of ferroptosis. PRMT9 methylates heat shock protein family A member 8, reducing oxidative stress levels and thereby inhibiting ferroptosis^148^. Additionally, studies have demonstrated that PRMT4 inhibits the Nrf2/GPX4 pathway by asymmetrically dimethylating arginine residues in the Neh4-6 domain of Nrf2. This modification restricts the nuclear translocation of Nrf2, thereby suppressing the expression of GPX4. Consequently, ferroptosis is induced, which in turn promotes the progression of doxorubicin-induced cardiomyopathy^159^.

Research on histone methylation and non-histone methylation in ferroptosis has made certain progress. However, the roles and interrelationships of histone methylation and non-histone methylation in regulating the ferroptosis pathway, as well as the mechanisms by which methylated histones or non-histones, especially ferroptosis-related proteins, participate in the regulation of ferroptosis, still require further in-depth investigation.

### 2.6. Lipidation

Lipidation is a protein modification that targets proteins to organelles, vesicles, and plasma membranes. The association between this protein modification and ferroptosis is not fully understood. A distinctive feature of ferroptosis is the generation and buildup of lipid hydroperoxides, particularly the oxidized derivatives of polyunsaturated PE. These compounds are susceptible to iron-catalyzed secondary radical reactions, yielding products that maintain the characteristic PE headgroup and can interact with nucleophilic regions of proteins via truncated electrophilic acyl chains. Consequently, investigators have employed redox lipidomics techniques to detect oxidized truncated PE species in both enzymatic and non-enzymatic model systems. Identifying PE LPO adducts on proteins can help find new biomarkers for ferroptosis^160^. The lipidation of light chain protein-3 (LC-3) is associated with ferroptosis to a certain extent. For example, under type II inflammatory conditions, 15-lipoxygenase-1 (15-LO1) in human airway epithelial cells (HAECs) forms a 15-LO1-PEBP1 complex with PEBP1. The complex generates iron-avid hydroperoxyeicosatetraenoyl phosphatidylethanolamines (HpETE-PEs) that lipidate the autophagy protein microtubule light chain protein-3I (LC-3I), forming membrane-bound lipidated LC3-II. This process stimulates the initiation of protective autophagy, allowing cells to escape ferroptosis and the release of mitochondrial DNA. The balance between the 15-LO1-PEBP1 complex and lipidated LC3-II is conducive to targeted therapy and improving asthma prognosis^161^. In retinal pigment epithelial (RPE) cells, the complex formed by lipocalin 2 (LCN2) binding with ATG4B to form the LCN2-ATG4B-LC3-II complex regulates the activity of ATG4B and the lipidation of LC3-II, reducing autophagic flux. Moreover, due to the correlation between the activation of the inflammasome and the increased fluorescence intensity of the iron death marker Liperfluo dye in live RPE cell imaging, it indicates that oxidative stress induces ferroptosis. This study identifies an increase in LCN2 as a pathophysiological trigger in RPE, also providing a therapeutic approach in the context of ferroptosis^162^. Additionally, the extensive lipidation of the autophagy marker protein LC3B in GBM cells is induced by three compounds: loperamide, pimozide, or STF-62247, with ferroptosis also playing a certain secondary role^163^. Lipidation modifications, which are covalent attachments of lipids to proteins, endow proteins with distinct membrane affinities. Concurrently, a single protein may undergo multiple lipidations^164^. The repertoire of lipid moieties that can be attached to proteins through lipidation includes, but is not limited to, fatty acids (FA), sulfate, isoprenoids, sterols, phospholipids, glycosylphosphatidylinositol (GPI) anchors, and lipid-derived electrophilic species. Categorization of protein lipidation is predicated on the subcellular locale of the modification: the first category encompasses modifications occurring in the cytoplasm or on the luminal side of the plasma membrane, including S-palmitoylation, N-myristoylation, and S-prenylation. The second category pertains to modifications that transpire within the confines of the secretory organelle lumen, exemplified by GPI anchoring and cholesterolation^165^. The distinct lipidation processes of N-myristoylation, palmitoylation, and S-prenylation are implicated in the modulation of ferroptosis, suggesting a mechanistic interplay between protein lipidation and cellular susceptibility to this form of regulated cell death.

#### 2.6.1. N-myristoylation

N-myristoylation (MYR) is a lipid modification of proteins that facilitates the binding of proteins to the cell membrane. Myristoylated proteins can stably attach to or reversibly detach from the cell membrane. The conjugation of the myristoyl moiety to the glycine residue in a protein following translation is mediated by N-myristoyltransferase (NMT) and myristoyl-CoA. Traditionally, N-myristoylation was believed to occur exclusively on N-terminal glycine residues; however, recent findings have revealed that N-myristoylation can also occur on the ε-amino group of internal lysine residues. In the presence of both α- and ε-amino acids within the same peptide segment, NMT exhibits a preference for catalyzing the myristoylation of Gly over Lys. Contrasting the irreversible nature of glycine myristoylation, the identification of various acyl hydrolases that specifically target Lys myristoylation has been reported,including Sirtuin1/2/3/6 (SIRT1/2/3/6) and histone deacetylases 8/11 (HDAC8/11), indicates that Lys myristoylation is a reversible modification. This suggests that, like other reversible modifications such as palmitoylation and phosphorylation, lysine myristoylation may also be intimately involved in the regulation of various signaling pathways, potentially including those involved in ferroptosis^164^. FSP1, as a member of the N-myristoylated (N-MYR) protein family, is localized to the plasma membrane and functions as a redox enzyme, converting ubiquinone to ubiquinol to inhibit ferroptosis. Although FSP1 is considered a parallel system to GPX4, in plants, it has been discovered that among the eight GPX isoforms, GPX4/5 belong to the category of N-MYR proteins. Therefore, GPX4/5 may also exert a similar N-MYR protein function to FSP1/AIFM2 at the plasma membrane^166^. The process of ferroptosis, marked by rampant LPO culminating in a controlled cell demise dubbed ferroptotic necrosis, is linked to cancer cell metastasis. The formation of ovarian cancer spheroids and exposure to platinum-based chemotherapy both increase the levels of anti-ferroptotic proteins and ACSL1. The upregulation of ACSL1 reduces the levels of LPO, thereby enhancing the cells' resistance to ferroptosis. FSP1 as a potential candidate for N-myristoylation, further augments ferroptosis resistance in an ACSL1-enhanced manner. This suggests that ACSL1 modulates the N-myristoylation of FSP1 to bolster antioxidant capacity and increase resistance to ferroptosis^34^. In the pathogenesis of excitotoxicity in neurodegenerative diseases, although the role of ferroptosis is not fully understood, some studies have used transcriptomic analysis to show that reduced levels of NADPH in the cytoplasm are associated with susceptibility to compounds that induce ferroptosis. Moreover, exogenous NADPH interacts with NMT2, upregulating N-myristoylated FSP1. NADPH increases the membrane localization of FSP1, enhancing resistance to ferroptosis. This indicates that NMT2 is essential in correlating NADPH levels with neuronal vulnerability to ferroptosis^37^.

#### 2.6.2. Palmitoylation

Palmitoylation is a reversible form of lipidation in which a saturated fatty acid, palmitic acid, is enzymatically attached to a free cysteine residue near the protein-lipid interface, forming a thioester linkage with the cysteine residue^37^. This process is typically catalyzed by members of the zinc finger, aspartate-histidine-histidine-cysteine (ZDHHC) palmitoyltransferase family^167^. The highly dynamic nature of palmitoylation contrasts significantly with the relatively static nature of similar lipid modifications, such as myristoylation^168^. The unique reversibility of protein palmitoylation permits rapid translocation of proteins between cellular membranes. The palmitic acid cycle can also be modulated by various physiological stimuli, contributing to cellular homeostasis and plasticity^169^. Inhibitors of palmitoyl protein thioesterase 1 (PPT1), such as DC661, suppress the induction of lysosomal LPO, leading to lysosomal membrane permeabilization and the induction of ferroptosis, with lysosomes being one of the primary storage sites for iron. Following treatment with DC661, the transcriptional levels of three hallmarks of ferroptosis—prostaglandin-endoperoxide synthase 2, cationic amino acid transporter 1, and cysteine-tRNA synthetase—are upregulated. However, it is intriguing that the inhibition of ferroptosis does not ameliorate the cytotoxicity associated with DC661^170^. Recent research has indicated that while targeting PPT1 alone may not be sufficient for the treatment of melanoma, modulating ferroptosis and antioxidant pathways could potentially offer a strategy to enhance therapeutic efficacy^171^. Additionally, carnitine palmitoyltransferase 1A (CPT1A), identified as a pivotal rate-limiting enzyme in fatty acid oxidation through metabolomic, transcriptomic analyses, lung epithelial-specific CPT1A knockout mouse models, and clinical studies, synergizes with l-carnitine from tumor-associated macrophages to promote ferroptosis resistance and CD8^+^ T cell inactivation in lung cancer. Inducing ferroptosis in lung cancer cells and synergizing with immunotherapy has been demonstrated^172^.

S-palmitoylation is an essential protein modification required for the stability of the SLC7A11 protein. In GBM, SLC7A11 undergoes S-palmitoylation, which is catalyzed by ZDHHC8, a protein acyltransferase (PAT) family member, specifically at the Cys327 residue, which consequently diminishes the ubiquitination of SLC7A11. AMPKα1 directly phosphorylates ZDHHC8 at the S299 site, thereby augmenting the interaction between ZDHHC8 and SLC7A11, facilitating S-palmitoylation and deubiquitination of SLC7A11. These findings underscore that in GBM tumors, the ferroptosis resistance mediated by ZDHHC8-driven SLC7A11 S-palmitoylation represents a novel therapeutic strategy for GBM^18^. The enhancement of DUXAP8 is closely associated with the malignant phenotype of HCC cells. DUXAP8 regulates the palmitoylation levels and protein stability of SLC7A11. It has been found that DUXAP8 can augment the palmitoylation of SLC7A11. Research indicates that DUXAP8 participates in the process of ferroptosis through the mediation of SLC7A11. The downregulation of DUXAP8 increases the production of MDA, the ratio of oxidized glutathione to reduced glutathione (GSSG/GSH ratio), and the levels of ROS in HCC cells induced by sorafenib and erastin. Silencing DUXAP8 may enhance ferroptosis in tumor cells. The inhibitory effect of DUXAP8 overexpression on ferroptosis can be reversed by the knockdown of SLC7A11. Therefore, the long non-coding RNA (lncRNA) DUXAP8, by acting on the SLC7A11-mediated ferroptosis pathway, may achieve higher anti-hepatoma efficacy when used in combination with sorafenib^173^. In CRC, the interferon-gamma (IFNγ) receptor (IFNGRs) and Janus kinase/STAT1 can modulate the IFNγ signaling pathway through modifications such as palmitoylation. The downregulation of the two subunits of system Xc-, SLC3A2 and SLC7A11, impairs the tumor cells' cystine uptake, promoting LPO and ferroptosis in tumor cells. In CRC cells, IFNGR1, an essential component of the IFNγ signaling pathway, can undergo palmitoylation, which facilitates the degradation and instability of IFNGR1 in CRC cells. Targeting the stability of IFNGR1 holds promise to overcome the resistance of CRC patients to immune checkpoint blockade^174^. Recent studies have demonstrated that GPX4, a pivotal anti-ferroptosis protein, undergoes ZDHHC20-mediated palmitoylation at the Cys66 residue, which stabilizes the protein and mitigates ferroptosis. Inhibition of the depalmitoylase APT2 enhances GPX4 palmitoylation, thereby promoting resistance to ferroptosis. Conversely, disruption of GPX4 palmitoylation exacerbates ferroptosis, suppresses tumor metastasis, and impacts ischemia-reperfusion injury. Deciphering the GPX4 palmitoylation network may provide innovative therapeutic approaches for diseases associated with ferroptosis^175^. The palmitoylation of STING represents an effective pharmacological target for the inhibition of STING signaling, with research suggesting its potential utility in the treatment of STING-dependent pathologies^176^. The cGAS-STING pathway is one of the mechanisms through which ferroptosis can occur, thus a potential correlation between STING palmitoylation and ferroptosis may exist.

To summarize, palmitoylation serves as a precise regulatory mechanism for the behavior and function of cellular proteins, especially those implicated in membrane-associated processes, exemplified by the regulation of cysteine uptake via SLC7A11-mediated cellular membrane processes^9^.

#### 2.6.3. S-prenylation

Initially documented in fungi in 1978 and later in mammals, S-prenylation denotes the covalent linkage of either a 15-carbon farnesyl or a 20-carbon geranylgeranyl moiety to protein cysteine residues through a thioether bond^177^. This process is primarily catalyzed by protein farnesyltransferase (FTase) or protein geranylgeranyltransferase type I (GGTase-I)^178^. S-prenylation is an important drug target, with known substrates including small GTPases of the Ras, Rho, and Rab families, nucleolar proteins, as well as kinases and phosphatases^179^. To date, four heterodimeric prenyltransferases are known to be responsible for protein prenylation: protein farnesyltransferase (FTase), protein γ-glutamyltransferase type I (GGTase-I), Rab geranylgeranyltransferase (GGTase-II), and protein γ-glutamyltransferase type III (GGTase-III). Biochemical analyses have demonstrated that a considerable subset of prenylated proteins harbor a C-terminal CAAX motif, which serves as a recognition site for enzymatic modification. Within this motif, "C" denotes a cysteine residue, "A" typically represents a hydrophobic amino acid, and "X" specifies any amino acid that determines the prenylation type, either farnesylation or geranylgeranylation. CAAX proteins generally undergo three successive stages: initial prenylation, proteolytic processing, and terminal carboxymethylation, before translocating to the plasma membrane^178^. Studies have demonstrated that the selective targeting of geranylgeranylation of certain guanine nucleotide-binding proteins serves as a significant model for investigating substrate/geranylgeranyltransferase I interactions, illustrating the feasibility of altering the prenylation status of proteins within living cells^180^. Terpenoid natural products, synthesized through cyclization or prenylation reactions based on carbocations, have also been shown to possess the capability to catalyze C-, N-, O-, and S-prenylation on small molecules^181^. In the context of statin research, their antitumor effects can be attributed to the disruption of protein prenylation, reduction of cholesterol levels, modulation of autophagy, promotion of ferroptosis in tumor cells, and inhibition of their proliferation and invasion^178^.

### 2.7. Glycosylation

Glycosylation is a prevalent form of PTM. It refers to the process by which carbohydrate chains, under the influence of enzymes, are attached to proteins and lipids. These carbohydrate chains, transferred to proteins by glycosyltransferases, form glycosidic bonds with amino acid residues, resulting in the formation of glycoproteins^182^. The majority of nuclear and cytoplasmic proteins undergo O-linked β-N-acetylglucosamine (O-GlcNAc) glycosylation, a post-translational modification where β-N-acetylglucosamine is enzymatically attached to the hydroxyl group of serine or threonine residues on the protein backbone^183^. Proteins are glycosylated through a complex network of metabolic pathways and various glycosylation processes.

Cigarette smoke (CS) can upregulate the levels of iron within corneal epithelial cells by increasing the expression of TFRC and promote LPO by increasing the expression of ACSL4. Exposure to CS increases the activity of O-GlcNAc transferase, leading to the O-GlcNAcylation of YAP, which induces ferroptosis in corneal epithelial cells^184^. Studies have found that the dynamic modification of O-GlcNAcylation drives ferroptosis by coordinating ferritinophagy and mitophagy. Ferroptosis inducers, such as RSL3, lead to the removal of O-GlcNAc from serine 179 of the ferritin heavy chain, which increases its binding to the ferritin receptor NCOA4. This modification results in the accumulation of labile iron within the mitochondria, leading to iron-induced instability and cell death^185^. ZIP8, a metal ion transporter, is vital for RPE degeneration by facilitating zinc and iron transport. The modification at N-glycosylation sites N40, N72, and N88 is essential for ZIP8-mediated intracellular iron accumulation, which further results in elevated LPO and subsequent RPE cell death^186^.

Glycosylation appears to have a significant impact on overcoming tumor drug resistance. In pancreatic ductal adenocarcinoma (PDAC), the glycosyltransferase B3GNT3 can catalyze the glycosylation of 4F2hc (SLC3A2), stabilize the 4F2hc protein, and enhance the interaction between 4F2hc and xCT. Treatment with the classic N-glycosylation inhibitor tunicamycin (TM) significantly triggers excessive activation of LPO and enhances the sensitivity of PDAC cells to ferroptosis^20^. ALG3, an α-1,3-mannosyltransferase, is implicated in protein glycosylation within the endoplasmic reticulum (ER). Inhibition of ALG3 or treatment with TM synergizes with anti-PD1 therapy to inhibit tumor growth in mouse cancer models. Inhibition of ALG3 induces defects in post-translational N-linked glycosylation modifications and leads to excessive lipid accumulation in cancer cells through sterol regulatory element-binding protein (SREBP1)-dependent lipogenesis, inducing immunogenic ferroptosis in cancer cells and promoting a pro-inflammatory microenvironment, thereby enhancing the antitumor immune response^187^. In HCC, targeting USP8 can promote ferroptosis, leading to reduced stability of OGT, which in turn inhibits the O-GlcNAc glycosylation of SLC7A11^188^.

Further investigation is required to elucidate the specific proteins involved in ferroptosis that are targeted by glycosylation and the pathways through which glycosylation regulates ferroptosis.

### 2.8. S-Nitrosylation

S-nitrosylation is a PTM^189^ involving the covalent binding of NO to proteins, and it is closely associated with cell death. The S-nitrosylation of proteins is a reversible redox process. NO, a lipophilic and highly diffusible radical signaling molecule, has been implicated in inducing apoptosis in tumor cells through S-nitrosylation. Studies have shown that, on one hand, NO has tumor-promoting effects at measurable concentrations; on the other hand, it appears to be cytotoxic to a variety of tumors. Research suggests that it could serve as a novel cancer treatment method. Numerous studies have indicated that the inhibition of NO can activate ferroptosis. Additionally, NO possesses an S-nitrosylation regulatory mechanism that is independent of the classical NO/sGC/cGMP signaling pathway^190^. Due to the instability and low abundance of NO, it predominantly undergoes S-nitrosylation with specific molecules or is converted into derivatives. Apart from direct reactions, S-nitrosylation is primarily achieved through trans-nitrosylation due to the instability of NO. The main small molecule nitrosylating agents within cells are S-nitrosoglutathione (GSNO), which typically act as a reservoir for NO, responsible for its transport and storage *in vivo*^191^. Furthermore, reactive nitrogen species (RNS)^192^ are also associated with the S-nitrosylation of intracellular target proteins. Studies have demonstrated that inflammation induces oxidative stress through various ROS and RNS. Peroxynitrite is generated via the chemical reaction between O2• and •NO, leading to oxidative damage, or reacting with tyrosine residues or thiol-containing proteins to form 3-nitrotyrosine or reversible thiol S-nitrosylation. Cancer cells experience persistent oxidative stress, delicately balancing iron and thiols to avert ferroptosis^193^. In DCM, the upregulation of mitogen-activated protein kinase kinase kinase kinase 4 (MAP4K4) induces S-nitrosylation of Drp1 (SNO-Drp1) and suppresses GPX4 expression, culminating in mitochondrial dysfunction and endothelial cell ferroptosis, which exacerbates cardiac microvascular damage. In the db/db mouse model, inhibiting MAP4K4 or mutating the C644 site of Drp1 mitigates cardiac microvascular injury and cardiac dysfunction, highlighting MAP4K4 and SNO-Drp1 as potential therapeutic targets for DCM^194^.

Current tumor treatment strategies involving NO are designed to induce cell death by promoting apoptosis via S-nitrosylation. However, various shortcomings have hindered this form of tumor treatment. Utilizing the pathway of ferroptosis may be a more effective anti-tumor treatment method. Numerous studies have indicated that cellular S-nitrosylation levels are controlled by both S-nitrosylation and denitrosylation processes. The denitrosylation process is primarily regulated by two enzymatic systems: thioredoxin/thioredoxin reductase (Trx/TrxR) and S-nitrosoglutathione reductase (GSNOR). An NO donor, NOC18, can inhibit ferroptosis induced by various treatments, but it does not alter the iron status during the ferroptosis process. Alternatively, it inhibits ferroptosis by interrupting the LPO chain reaction, indicating that NO concentrations significantly influence LPO during ferroptosis. Moreover, S-nitrosylation modulates the P38/JNK, MAPK, and Nrf2 signaling pathways implicated in ferroptosis^191^.

### 2.9. Oxidative Modification

Dysregulation of ROS and ROS-regulated signaling pathways has a significant impact on the occurrence, growth, and progression of cancer. The herbal compounds Quercetin, Acteoside, Silybin, and Daphnetin can all downregulate ROS levels to a certain extent^195^. ROS, including hydrogen peroxide (H_2_O_2_), can directly oxidize protein thiols, resulting in the formation of sulfenic (RSO_2_H) and sulfonic (RSO_3_H) oxidation states. This PTM can lead to altered protein function^196^. Regarding target molecules, singlet oxygen (^1^O_2_) reacts with unsaturated fatty acids, leading to LPO. Enzymes with active cysteine groups in their catalytic centers are susceptible to the effects of ^1^O_2_^197^. Studies have found that ROS can affect the function of various proteins involved in the epithelial-mesenchymal transition (EMT) process by reversibly or irreversibly oxidizing free cysteine residues on proteins^198^. Oxidative stress is caused by the excessive accumulation of ROS without effective neutralization by antioxidants. In the process of ROS detoxification, GSH resists the occurrence of ferroptosis^199^. Furthermore, in the cytoplasm, excessive iron levels promote oxidative stress, leading to an uncontrolled surge in calcium signaling, which damages mitochondrial function. Dysregulation of iron homeostasis causes an increase in cytosolic calcium levels, resulting in increased mitochondrial calcium, intensified oxidative stress, and damage. This leads to neuronal dysfunction and death^200^. Emerging research indicates that intracellular bicarbonate anions modulate the Fenton reaction, reducing oxidative damage and DNA repair burden, offering new insights into cellular defense against oxidative stress^201^. PRDX3, as a marker of ferroptosis in chronic liver disease, is peroxidized by mitochondrial lipid peroxides, converting cysteine thiols to sulfinic or sulfonic acids while translocating itself to the plasma membrane, inhibiting cystine uptake, and subsequently leading to ferroptosis^19^.

### 2.10. Lactylation

Lactylation, a product of the Warburg effect, is traditionally considered a byproduct associated with energy and metabolism, and the Warburg effect is implicated in a variety of cellular physiological and pathological processes. The Warburg effect, first discovered by Otto Warburg in the 1920s, refers to the phenomenon that cancer cells tend to increase glycolysis to ferment glucose into lactate even under aerobic conditions, rather than generating energy through mitochondrial oxidative phosphorylation^202^,^203^. The presence of functionally normal mitochondria in most cancer cells suggests that the Warburg effect may be a consequence of mitochondrial activity becoming saturated or even overloaded. This condition drives cancer cells to reprogram their metabolism, fermenting excess glucose into lactate to meet the demands for energy and biosynthesis required by rapid proliferation^204^. Cancer cells increase the expression of glucose transporters (GLUTs) to enhance glucose uptake^203^, glucose is metabolized in the cytoplasm through a series of enzyme-catalyzed reactions to generate pyruvate^205^. Subsequently, pyruvate is reduced to lactate by lactate dehydrogenase (LDH) and then transported out of the cell via monocarboxylate transporters (MCTs)^203^,^204^. Also, lactate dehydrogenase A (LDHA) can modulate the progression of ferroptosis in VSMCs by downregulating the expression of Nrf2, thereby emerging as a potential therapeutic target for AD^206^. Current research on the role of lactate in physiology and disease is not yet clear. Histone lysine lactylation, derived from lactate, is a novel post-translational epigenetic modification. Experimental evidence has shown that exogenous lactate can induce histone lysine lactylation, and this PTM can also occur through endogenous lactate, which originates from glucose within the body. The level of lactate depends on the balance between glycolysis and mitochondrial metabolism, and the activity of enzymes in both pathways can regulate lactate levels, thereby modulating histone lysine lactylation. Endogenous lactate production is a key determinant of histone lysine lactylation levels^22^. Hyperlactatemia exacerbates ferroptosis in hepatic IRI by activating lysine acetyltransferase 8 (KAT8), which lactylates phosphoenolpyruvate carboxykinase 2 (PCK2) at Lys100, thereby enhancing its kinase activity. PCK2, in turn, competitively inhibits Parkin-mediated polyubiquitination of 3-oxoacyl-ACP synthase, leading to metabolic remodeling of mitochondrial fatty acid synthesis and promoting ferroptosis^207^. In sepsis, the elevation of serum lactate levels exhibits a positive correlation with the associated mortality rates. Lactate can induce ferroptosis in sepsis-associated acute lung injury (ALI) both *in vivo* and *in vitro*. However, the ferroptosis inhibitor ferrostatin-1 can reverse the lactate-induced increase in Fe^2+^ and ROS levels in the MLE12 cell line, as well as the time-dependent downregulation of GPX4. Lactate modulates the level of N^6^-methyladenosine (m^6^A) modification by enhancing the binding of lysine 18 on histone H3 acetylated by lysine acetyltransferase p300 (H3K18ac)^208^ to the METTL3 promoter site. Under the stimulation of lactate, the METTL3/YTHDC1 axis mediates the stability of ACSL4 mRNA through m6A modification, which leads to an increase in ACSL4, inducing ROS accumulation and mitochondrial-dependent ferroptosis, thereby exacerbating lactate-induced ferroptosis in pulmonary epithelial cells^209^. Histone deacetylase inhibitors (HDACi) attenuate HDAC1K412 lactylation and FSP1 mRNA m^6^A modifications, thereby diminishing FSP1 levels and augmenting CRC vulnerability to ferroptosis^210^. Conversely, tectorigenin (TEC) leverages tRF-31R9J to recruit HDAC1, which in turn reduces histone lactylation and acetylation of ferroptosis-related genes, inhibiting hepatocyte ferroptosis and ameliorating non-alcoholic fatty liver disease^211^. The traditional Chinese medicine component Evodiamine inhibits the expression of HIF1A histone lactylation in prostate cancer (PCa) cells, significantly blocking lactate-induced angiogenesis. It further enhances the transcription of the signaling protein Sema3A, suppresses the transcription of PD-L1, and induces ferroptosis through the expression of GPX4, which can be reversed by ferrostatin-1^212^. Regarding the osteogenic differentiation of human bone marrow stem cells, the crystallin protein alpha B (CRYAB) acts as a molecular chaperone and plays an important role in the biological process of protein folding. It interacts with FTH1 to regulate the stability of the FTH1 protein in a lactylation-dependent manner. The downregulation of CRYAB promotes the degradation of FTH1, elevates the intracellular levels of Fe and ROS, ultimately promoting ferroptosis in bone marrow mesenchymal stem cells and slowing down their osteogenic differentiation^213^.

Lactylation modification is intricately linked to ferroptosis in the pathogenesis and progression of various diseases. Hyperlactatemia, through the activation of KAT8, induces lactylation of PCK2, thereby enhancing its kinase activity. This modification inhibits Parkin-mediated polyubiquitination of 3-oxoacyl-ACP synthase, leading to metabolic reprogramming of mitochondrial fatty acid synthesis. Consequently, this process promotes hepatocyte ferroptosis and exacerbates liver IRI.During sepsis, high serum lactate levels induce ferroptosis in lung tissue, which can be effectively reversed by ferrostatin-1. HDACi enhance the sensitivity of CRC to ferroptosis by attenuating lactylation of HDAC1 at lysine 412 and m6A modification of FSP1 mRNA.TEC utilize tRF-31R9J to recruit HDAC1, thereby reducing histone lactylation and acetylation of ferroptosis-related genes. This mechanism inhibits hepatocyte ferroptosis and ameliorates non-alcoholic fatty liver disease. The traditional Chinese medicine component Evodiamine can inhibit histone lactylation of HIF1A in PCa cells, thereby blocking angiogenesis and inducing ferroptosis in cancer cells. In contrast, CRYAB regulates the stability of FTH1 protein in a lactylation-dependent manner, thereby inhibiting ferroptosis in bone marrow mesenchymal stem cells. Lactylation modification is closely related to the Warburg effect, which is a key aspect of endogenous lactate. The Warburg effect, on one hand, inhibits mitochondrial respiratory chain complexes I and III, thereby suppressing the electron transfer of DHODH and destabilizing the mitochondrial membrane. On the other hand, the accumulation of lactate alters the intracellular NAD+/NADH ratio, increasing oxidative stress. Both mechanisms can activate ferroptosis^202^.

### 2.11. Interaction and Regulation of PTMs in Ferroptosis Targets

In the regulation of ferroptosis, different PTMs can be interconnected and influence each other through specific proteins as targets. PTMs do not function in a parallel or isolated manner, but rather form an intricate and sophisticated PTM network that exerts precise regulation of ferroptosis. Among these, the Ser42 site of GPX4, a key regulator of ferroptosis, can be phosphorylated by PKC. This phosphorylation alters its conformation, enhances its binding capacity to GSH, and simultaneously restricts the recognition by the E3 ubiquitin ligase TRIM26, thereby augmenting the antioxidant effect and inhibiting ferroptosis. In contrast, the K48 site of GPX4 can be recognized by TRIM26 and undergo ubiquitination and degradation. This process also reduces the phosphorylation sites of GPX4, downregulates the level of GPX4, and promotes ferroptosis. The deubiquitinase USP35 can remove the ubiquitination modification of GPX4, stabilize its level, and inhibit ferroptosis^214^. In Perioperative Neurocognitive Disorder, the acetylation level of GPX4 is elevated due to the downregulation of SIRT3. This results in decreased GPX4 activity and increased accumulation of ROS, thereby promoting ferroptosis^215^. Similarly, in kidney cells exposed to cadmium background, SIRT3 levels were downregulated, GPX4 acetylation levels were upregulated, and lipid peroxidation levels were upregulated, inducing ferroptosis^137^. Both acetylation and ubiquitination of GPX4 can destabilize the protein, leading to decreased activity and thereby promoting ferroptosis. This suggests that these two post-translational modifications (PTMs) may have a competitive relationship, where an increase in the level of one PTM may reduce the ability of the other PTM to recognize and target GPX4. Under oxidative stress, GPX4 can experience decreased activity due to oxidative modifications at its active sites, such as selenocysteine (Sec). Interestingly, the conformational changes in GPX4 caused by oxidative modifications enhance the recognition efficiency of TRIM26. Oxidatively modified GPX4 is subject to ubiquitination and degradation, reducing its accumulation within the cell, thereby lowering the level of oxidatively modified GPX4. However, the overall downregulation of GPX4 increases cellular sensitivity to ferroptosis^216^. The upstream regulator of GPX4, SLC7A11, can also be recognized at its K48 site by the E3 ubiquitin ligase CRL3KCTD10 and undergo ubiquitination and degradation. This process downregulates its transport capacity and promotes ferroptosis^217^. Specific lysine residues of SLC7A11 can be recognized by PIAS4 and subjected to SUMOylation. This modification alters the conformation of SLC7A11, thereby enhancing its stability and potentially restricting the recognition by E3 ubiquitin ligases, thus inhibiting ferroptosis^98^. Nrf2, an antioxidant transcription factor closely related to ferroptosis, undergoes a conformational change when its Ser40 site is phosphorylated by PKC. This modification reduces the binding capacity of Kelch-like ECH-associated protein 1 (Keap1) to Nrf2, downregulates the ubiquitination and degradation of Nrf2, enhances the stability of Nrf2, and thereby inhibits ferroptosis. Similarly, Nrf2 can be acetylated by acetyltransferases, which lowers its ubiquitination level, stabilizes Nrf2, and inhibits ferroptosis. Conversely, multiple lysine residues of Nrf2 are recognized by Keap1, leading to its ubiquitination and degradation. This process downregulates the level of Nrf2, thereby reducing its phosphorylation or acetylation levels and promoting ferroptosis^218^. In the pathways associated with ferroptosis, a single protein can be modified by multiple PTMs in various directions. These different PTMs can either synergistically cooperate or competitively antagonize each other, forming a complex PTM network that interdependently regulates the occurrence and progression of ferroptosis. However, the underlying logic between different PTMs within this network still requires further investigation.

## 3. The Therapeutic Potential of PTMs Targeting Ferroptosis-Related Proteins

In recent years, with the in-depth investigation of the mechanisms underlying ferroptosis, numerous clinical trial drugs related to ferroptosis or drugs approved by the FDA have been found to directly or indirectly modulate ferroptosis-related proteins via PTMs, thereby influencing disease progression. These drugs primarily exert their effects through the regulation of iron ion metabolism, lipid metabolism, and cellular redox metabolism.

### 3.1. Iron Metabolism Regulator

The core mechanism of ferroptosis involves free iron, and the regulation of free iron metabolism is a pathway through which many drugs exert their effects. The Ser687 residue of TFRC can be de-O-GlcNAcylated by erastin, reducing the binding of MARCH8 and decreasing polyubiquitination on Lys665, thereby promoting hepatocyte ferroptosis in the treatment of HCC^63^. The HECT-domain-containing ubiquitin E3 ligase HUWE1, acting as a negative regulator of ferroptosis, specifically TfR1 for ubiquitination and proteasomal degradation, thereby regulating iron metabolism, countering abnormal iron accumulation, and inhibiting ferroptosis, which may potentially alleviate acute liver injury caused by ischemia-reperfusion (I/R). Additionally, the membrane expression of TfR1 is positively regulated by PKC phosphorylation, which in turn promotes iron uptake. Deferoxamine (DFO) inhibits ferroptosis by chelating extracellular Fe³⁺, thereby weakening TfR1-mediated iron influx. This may be one of the mechanisms by which DFO inhibits myocardial cell ferroptosis in I/R^219^,^220^. The DMT1 transports Fe²⁺ into the cytoplasm, forming the LIP, which lays the foundation for the Fenton reaction. Histone deacetylase (HDAC) inhibitors, such as vorinostat (SAHA), induce high acetylation of DMT1, enhancing its membrane localization and Fe²⁺ influx, thereby sensitizing tumor cells to ferroptosis and increasing their sensitivity to chemotherapeutic drugs^221^. In the 6-OHDA Parkinson's disease model, FTH1 is ubiquitinated and degraded via NCOA4-mediated ferritinophagy, leading to the accumulation of free ferrous ions and triggering neuronal ferroptosis. Chloroquine (CQ) indirectly inhibits the ubiquitination and degradation of FTH1 mediated by NCOA4 by blocking the fusion of autophagosomes and lysosomes, thereby significantly alleviating ferroptosis-related neuronal damage^222^. FPN1 serves as the cellular "iron gate"—it pumps iron out of the cell, thereby determining the level of free iron within the cell. In lung cancer, the hepcidin-RNF217/DTX3L axis can lead to the ubiquitination and degradation of FPN1, resulting in iron retention and tumor growth. In contrast, USP35 stabilizes FPN1 by removing K48 ubiquitin chains, thereby inhibiting ferroptosis and promoting chemoresistance. "Blocking FPN1 degradation" (e.g., USP35 activators or RNF217/DTX3L inhibitors) may emerge as a new strategy to sensitize solid tumors such as lung and renal cancer to ferroptosis therapy. Conversely, "promoting FPN1 degradation" (e.g., hepcidin analogs) could be utilized for iron reduction therapy in conditions such as hemochromatosis, which is characterized by iron overload^223^,^224^. Iron metabolism regulatory drugs modulate the stability and localization of core ferroptosis proteins such as FPN1, FTH1, and TFR1 through PTMs including phosphorylation, ubiquitination, and acetylation, thereby rapidly reshaping the intracellular iron pool. These drugs have demonstrated therapeutic potential in various disease models, including myocardial ischemia, Parkinson's disease, lung cancer, and leukemia, by sensitizing chemotherapy, inhibiting ferroptosis, or reversing iron overload.

### 3.2. Regulators of Proteins Associated with Lipid Metabolism Pathways

In the context of cancer, the phosphorylation of ACSL4 emerges as a crucial node for enhancing ferroptosis and inhibiting tumor progression. For instance, upon sensing initial lipid peroxides, PKCβII phosphorylates and activates ACSL4, amplifying the peroxidation of PUFA-PLs, thereby synergistically inducing ferroptosis in tumor cells in combination with immunotherapy. Tamoxifen, by inhibiting the phosphorylation of ACSL4-Thr328 by PKCβII, attenuates PUFA-lipid synthesis, thereby increasing the resistance of breast cancer cells. This provides a repurposing strategy for combining tamoxifen with ferroptosis inducers such as RSL3 in triple-negative breast cancer with high ACSL4 expression^225^,^16^,^117^. Conversely, in prostate cancer, the phosphatase LHPP dephosphorylates ACSL4, thereby blocking its recognition by SKP2 and reducing ubiquitin-mediated degradation, thus stabilizing the ACSL4 protein. This stabilization similarly promotes ferroptosis and inhibits tumor growth^117^. In the context of myocardial injury, protocatechuic aldehyde (PrA) inhibits the phosphorylation of ACSL4, thereby blocking its mediation of phospholipid peroxidation. Additionally, PrA suppresses the autophagic degradation of FTH1, reducing the release of free iron. These actions collectively protect cardiomyocytes from ferroptosis^118^. In the spinal cord injury model, TRIM28 mediates the SUMOylation of ACSL4, thereby inhibiting its ubiquitination and degradation, enhancing protein stability, and promoting neuronal ferroptosis, which exacerbates tissue damage^226^. Metformin can activate AMPK, which in turn inhibits the phosphorylation of SCD1 at Ser318, leading to enzyme inactivation, reduced synthesis of MUFAs, and relative enrichment of PUFAs^227^. In advanced HCC patients, the unconventional prefoldin RPB5 interactor (URI) directly interacts with TRIM28 and promotes p53 ubiquitination and degradation in a TRIM28-MDM2-dependent manner. p53 binds to the promoter of SCD1 and suppresses its transcription. The combination of the SCD1 inhibitor arachidonyl trifluoromethyl ketone (ATF) and the deuterated sorafenib derivative donafenib has demonstrated promising antitumor effects in organoids and xenografted tumors derived from p53 wild-type HCC patients^228^.

In chemotherapy resistance of gastric cancer, cancer-associated fibroblasts (CAFs) package miR-522 into exosomes via the USP7/hnRNPA1 axis. These exosomal miR-522 molecules directly bind to the 3'UTR of ALOX15 mRNA, inhibiting its translation and thereby reducing the accumulation of ROS, which leads to cisplatin/paclitaxel resistance. Knockdown of miR-522 or inhibition of USP7 can restore ALOX15 expression and sensitize cells to ferroptosis^82^. In the context of acute kidney injury, SYK-mediated phosphorylation of ALOX15 at tyrosine sites enhances its catalytic activity, driving a burst of lipid ROS in renal tubules and promoting ferroptosis^229^. Additionally, the broad-spectrum PKC inhibitor GF109203X can reduce the phosphorylation of ALOX5AP, thereby decreasing leukotriene production and alleviating asthma airway inflammation^230^.

PTMs of key proteins such as ACSL4, SCD1, and ALOX15 (e.g., phosphorylation, SUMOylation) play crucial roles in tumor progression, chemoresistance, and tissue damage. These regulatory points offer potential therapeutic targets, such as combining ferroptosis inducers, inhibiting specific kinases, or modulating the activity of metabolic enzymes, which may improve chemoresistance and tissue damage.

### 3.3. Regulators of Proteins Associated with Antioxidant Pathways

Recent studies have revealed that the activity and stability of GPX4 are dynamically regulated by multiple layers of PTMs and have shown targetable intervention potential in the treatment of diseases such as cancer and organ damage. In terms of ubiquitination, the small molecule N6F11 specifically recruits the E3 ligase TRIM25, which catalyzes the polyubiquitination of lysine 48 (K48) on GPX4, leading to its proteasomal degradation. This selectively triggers ferroptosis in pancreatic cancer cells and enhances CD8⁺ T cell-mediated antitumor immunity. In contrast, the deubiquitinase USP8 stabilizes GPX4 by removing ubiquitin chains, thereby attenuating ferroptosis and potentially reducing the efficacy of immunotherapy^231^,^84^. In terms of palmitoylation, zDHHC8 catalyzes S-palmitoylation at the Cys75 site of GPX4, inhibiting its proteasomal degradation and maintaining its anti-ferroptotic function. The small molecule PF-670462 enhances the degradation of zDHHC8, thereby weakening this modification, significantly increasing tumor cell sensitivity to ferroptosis inducers, and synergizing with anti-PD-1 immunotherapy in a melanoma model^232^. Additionally, p53 recruits HDAC1 to deacetylate H3K27 in the GPX4 promoter region, thereby epigenetically silencing its transcription and enhancing the sensitivity of hepatocellular carcinoma cells to ferroptosis induced by artemisinin derivatives. Vorinostat (an HDAC1 inhibitor) can reverse this silencing, restore GPX4 expression, and alleviate ferroptosis^233^. Glycosylation is equally critical. N-glycosylation maintains the proper folding and membrane localization of GPX4. Its absence results in a decrease in enzyme activity by more than 50%, thereby exacerbating lipid peroxidation damage in ischemia-reperfusion myocardial cells^234^. Additionally, the therapeutic potential of SLC7A11 and Nrf2, which are upstream of GPX4 and subject to multiple PTMs, is also evident in various diseases. The AR recruits the E3 ubiquitin ligase NEDD4L to ubiquitinate and degrade SLC7A11, thereby inhibiting ferroptosis and contributing to castration-resistant prostate cancer (CRPC) resistance to enzalutamide. Blocking the AR-NEDD4L axis or knocking out NEDD4L can reverse this resistance^235^. In spinal cord injury, neuronal p53 is upregulated post-injury, which transcriptionally activates the E3 ubiquitin ligase KLHL4. KLHL4 catalyzes the K48-linked ubiquitination and degradation of SLC7A11, thereby reducing GSH synthesis, inducing ferroptosis, and exacerbating secondary injury. Administration of the p53 inhibitor Pifithrin-α or KLHL4 knockout can both enhance the stability of SLC7A11, reduce lipid peroxidation, and improve motor function scores^236^. Dimethyl fumarate (DMF) is an approved NRF2 activator that covalently modifies the Keap1 protein (primarily targeting the Cys151 site), thereby blocking the ubiquitination and degradation of NRF2 by the Keap1-Cul3 E3 ubiquitin ligase complex. This action promotes the nuclear translocation of NRF2 and activates the expression of antioxidant genes, ultimately inhibiting the ferroptosis process. In models of multiple sclerosis (MS), DMF mitigates iron death damage in the nervous system through this mechanism and has been approved for oral treatment of relapsing MS. Additionally, in AKI models, DMF has demonstrated potential to reduce tubular iron death markers in Phase II clinical trials^237^,^238^,^239^.

iFSP1, an inhibitor of FSP1, blocks TRIM21-mediated ubiquitination of FSP1 (K63-linkage), thereby disrupting the CoQ10-NAD(P)H antioxidant axis and enhancing the sensitivity of glioblastoma (GBM) cells to ferroptosis^9^. Similarly, PRR11i increases LPO and disrupts the mitochondrial morphology regulated by DHODH, thereby promoting ferroptosis in GBM cells and enhancing their chemosensitivity to temozolomide (TMZ)^42^.

In summary, PTMs regulate the activity of proteins such as GPX4, SLC7A11, Nrf2, FSP1, and DHODH through multiple mechanisms, thereby influencing cellular ferroptosis and disease progression. These modifications have shown significant therapeutic potential in diseases such as cancer, neurodegenerative disorders, and organ injury. Targeting these modification processes holds promise for the development of new therapeutic strategies, enhancing the efficacy of ferroptosis inducers and improving immunotherapy outcomes, thereby providing new theoretical basis and potential targets for the clinical intervention of related diseases.

## 4. Conclusion and perspective

Ferroptosis, an iron-dependent form of cell death marked by LPO, involves numerous proteins regulated by PTMs in various pathways during its process. Different types of PTMs modify the functional groups of ferroptosis-related proteins after mRNA transcription and translation, endowing these proteins with specificity and functionality that can either promote or inhibit the occurrence and development of ferroptosis.

Numerous studies have found that different types of PTMs are involved in regulating the classic antioxidant pathway of ferroptosis, namely the SLC7A11-GSH-GPX4 pathway. Ubiquitination, a PTM involving the binding of ubiquitin molecules to target substrates, is one such modification. BAP1 inhibits the expression of SLC7A11 through deubiquitination^85^, while SMURF2 polyubiquitinates the GSTP1 protein leading to its degradation^73^, promoting cellular ferroptosis. On the other hand, USP8 removes the K48 ubiquitination from GPX4 to stabilize it^84^, inhibiting cellular ferroptosis. Sumoylation, a type of ubiquitination-like modification, involves the combination of SUMO with substrate proteins. GPX4 or its upstream regulatory factor Nrf2, when sumoylated, can downregulate the intracellular ROS levels^95^, or SLC7A11 induced by SENP1 expression^68^, both of which can limit the development of ferroptosis. Phosphorylation, an important PTM, functions by transferring the γ-phosphate group from ATP or GTP to the substrate protein. In the AMPK pathway, the level of GPX4 is downregulated due to the depletion of SIRT3, which in turn inhibits ferroptosis^107^. In the Nrf2-SLC7A11-GPX4 pathway, USP20, on the one hand, enhances the stability of SLC7A11 by deubiquitination, and on the other hand, enhances its own stability through phosphorylation at the Ser residue, thereby conferring ferroptosis resistance to HCC cells^119^. In ferritinophagy, the downregulation of STAT3 leads to the downregulation of NCOA4, which in turn induces ferroptosis^115^. Acetylation involves the transfer of an acetyl group to the side chains of amino acids. The acetylation of P53 can inhibit the expression of SLC7A11^126^, or downregulate GPX4 levels, and the knockout of SIRT3^130^, all of which can promote ferroptosis. Methylation modification involves the transfer of a methyl group from an active methyl donor to other proteins. Histone modification involves not only acetylation and ubiquitination but also methyl group modifications. Lipidation modification is the process of targeting proteins to organelles, vesicles, and the plasma membrane. Among them, S-palmitoylation, N-myristoylation, and S-prenylation are related to ferroptosis. N-myristoylation primarily facilitates the binding of proteins to the cell membrane, while S-palmitoylation is mainly the binding of the saturated fatty acid palmitic acid to free cysteines near the protein-lipid interface^37^. The PPT1 inhibitor DC661 upregulates ferroptosis markers through LPO lysosomes. ZDHHC8 catalyzes the S-palmitoylation of SLC7A11, deubiquitination, and enhances the stability of SLC7A11. Interestingly, AMPKα1 can enhance the function of the aforementioned ZDHHC8 through phosphorylation^18^. In addition, statins can promote ferroptosis by disrupting protein prenylation^178^. Glycosylation modification is the process of attaching sugar chains to proteins and lipids. In HCC, targeting USP8 inhibits the O-GlcNAc glycosylation of SLC7A11, inducing ferroptosis^188^. S-nitrosylation modification is a PTM in which proteins covalently bind with NO, and it mediates the P38/JNK, MAPK, and Nrf2 signaling pathways of ferroptosis^191^.

In the second ferroptosis inhibition system, the NADPH-FSP1-CoQ10 pathway, PTMs are also present. Target proteins with K63-linked ubiquitination are localized to the plasma membrane, which is closely associated with the function of FSP1 in suppressing ferroptosis. Edaravone can inhibit the ubiquitination of FSP1, preventing the destruction of its resistance to ferroptosis^9^,^75^. The myristoylation motif at the N-terminus of FSP1 plays a role in localizing to the plasma membrane. In the DHODH-CoQH2 antioxidant pathway, the downregulation of DHODH and CoQH2, cisplatin-induced CoQH2 deficiency, severe LPO, and mitochondrial dysfunction along with SIRT3-like SUMOylation^99^. Further investigation is needed regarding the regulation of the GCH1-BH4 antioxidant pathway by PTMs.

Oxidative modifications within PTMs can alter protein functions, and under lactate stimulation, m6A modification mediates the stability of ACSL4 mRNA, promoting mitochondria-dependent ferroptosis through histone lactylation^209^, which are relatively understudied areas in the mechanism of ferroptosis. However, PTMs such as ubiquitination, ubiquitin-like modifications, and acetylation are reversible due to the presence of one or more proteases, endowing proteins with more diversity, functionality, and plasticity. Moreover, different types of PTMs do not exist in isolation but have a certain degree of interactivity and correlation, forming a potential network system of PTMs.

However, research on ferroptosis mediated by PTMs is still in its nascent stage, with numerous issues remaining elusive. Firstly, it is acknowledged that the epigenetic level primarily regulates the modification of specific amino acids within the tail features of histones that possess covalent PTMs, a process mediated by enzymatically active proteins. Nonetheless, an in-depth investigation into histone PTMs is still lacking, and the role of other epigenetic factors in modulating ferroptosis in diseases remains unknown. Secondly, it is imperative to elucidate whether PTMs influence multiple ferroptosis regulators and how these diverse PTMs synergize with various signaling pathways to regulate cellular vulnerability to ferroptosis. Furthermore, the specific mechanisms by which PTMs govern the expression of genes associated with ferroptosis in diseases are not yet clear, and it is necessary to explore whether each PTM exerts specific regulatory control over its expression. Thirdly, despite numerous studies confirming the importance of PTMs targeting ferroptosis-related proteins in tumors and other diseases, the detailed mechanisms and molecular pathways associated with ferroptosis require further investigation. Fourthly, it is necessary to ascertain whether ferroptosis inducers/inhibitors are related to small molecule drugs that regulate PTMs, whether they exhibit synergistic effects, and whether they can achieve superior therapeutic outcomes. Additionally, the actual efficacy of established PTMs in inducing or inhibiting ferroptosis targets in preclinical and clinical trials, such as their application in anti-tumor drugs and their role when combined with other therapeutic strategies, needs to be explored. The specificity of the mechanisms of potential new PTMs that may be discovered in the future in a variety of diseases and cell types, the necessity for more systematic and comprehensive research is underscored.

In summary, PTMs are pivotal in the pathogenesis of diseases including cancer, hepatic disorders, pulmonary conditions, and renal ailments. The network of PTMs in the context of ferroptosis provides an epigenetic perspective for targeting therapies related to ferroptosis-associated diseases. However, further research into the impact of epigenetic modifications and PTMs influence ferroptosis will bring more opportunities to understand the pathogenesis of these diseases, especially those that are difficult to cure, offering new insights to improve previous treatment methods.

## Figures and Tables

**Figure 1:**
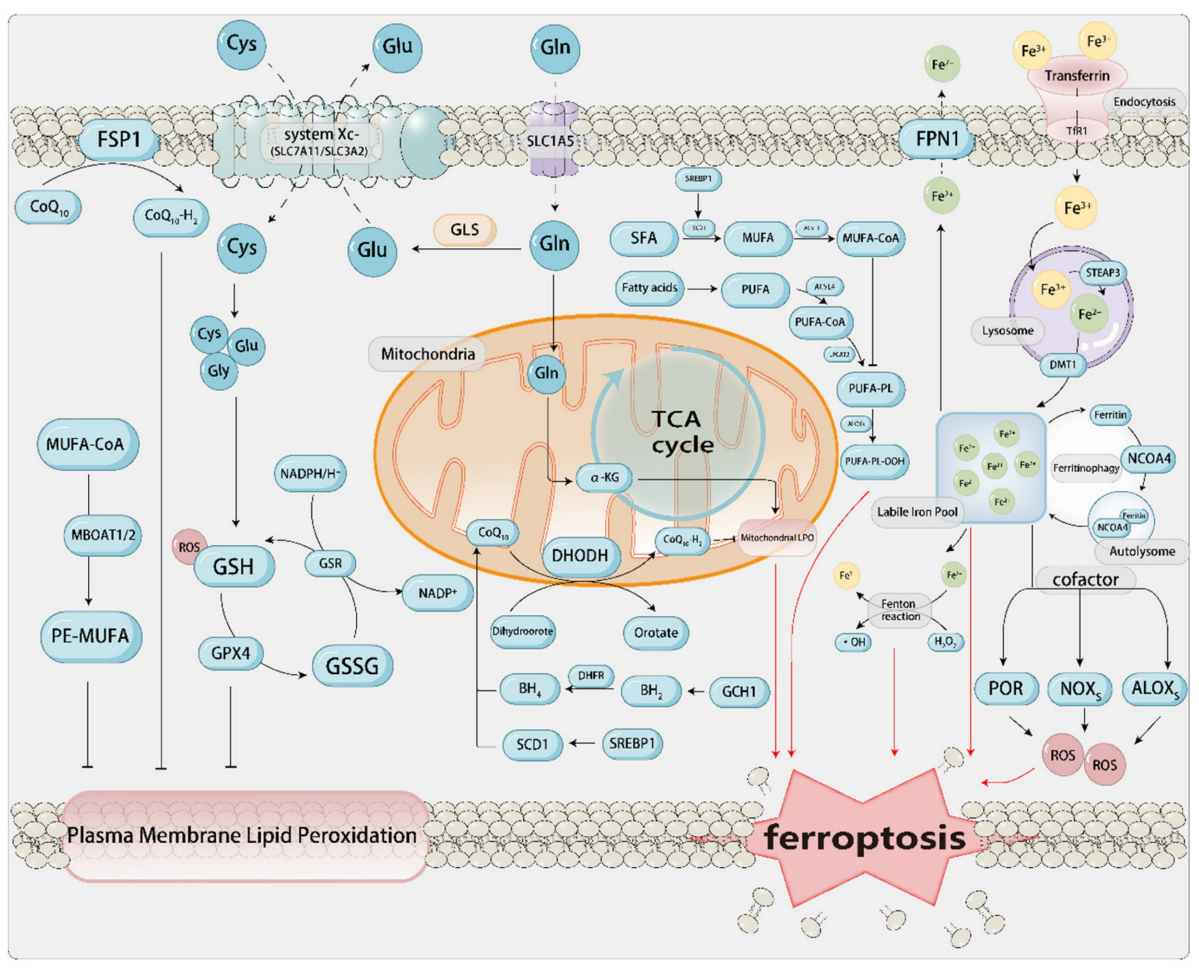
** The core mechanisms of ferroptosis and related antioxidant pathways. a. Iron metabolism** Fe^3+^ binds to TF and enters the cell via endocytosis through TFR, subsequently associating with lysosomes. Fe^3+^ dissociates from TF and is reduced to Fe^2+^ by STEAP3, then transported to the cytoplasm by DMT1, where it accumulates to form the LIP. Fe^2+^ from the LIP can be similarly stored in FT and transported to the mitochondria to support oxidative respiration, and it can also form autophagic lysosomes mediated by NCOA4. Fe^2+^ catalyzes the non-enzymatic Fenton reaction of PUFA-PLs, leading to the accumulation of lethal lipid peroxides, which triggers ferroptosis. It also acts as an essential cofactor for POR and ALOX to promote the progression of ferroptosis. Fe^2+^ can be exported to the extracellular space via FPN. **b. Lipid metabolism** PUFAs are catalyzed by ACSL4 and LPCAT3 to form PUFA-PLs, which are further oxidized by ALOX to produce PUFA-OOH, ultimately leading to the formation of LPO that induce ferroptosis. **c. Amino acid metabolism** The system Xc- (composed of SLC7A11 and SLC3A2) transports Cys and Glu into the cell. Glutamine enters the cell via SLC1A5 and is converted to Glu. Glu provides Cys for GSH synthesis through GCL. GPX4 utilizes GSH to reduce PL-PUFA-OOH to harmless products, preventing lipid peroxidation of the cell membrane. Similarly, the cell also has related antioxidant pathways, including the GPX4/xCT pathway, FSP1/CoQH2 pathway, DHODH/CoQH2 pathway, and GCH1/BH4 pathway, which can detoxify lipid peroxides and thus protect cells from ferroptosis damage.

**Table 1:** ** The mechanisms of various protein modifications in the context of disease-related ferroptosis.** Provides a comprehensive summary of the PTMs exerted by various enzymes on ferroptosis-related proteins, elucidating their mechanisms and potential associations with ferroptosis-related diseases.

Modification	Targets	Site	Enzyme	Disease	Mechanism	Ref.
Ubiquitination	GSTP1	K121/191/55	SMURF2	Cancer	SMURF2 primarily promotes the degradation of GSTP1 protein by catalyzing the polyubiquitination of GSTP1 at K121/191/55 sites, thereby promoting ferroptosis.	73
Ubiquitination	FSP1	K322/366	TRIM21	-	TRIM21 can promote the K63-linked ubiquitination of FSP1 at K322 and K366 sites, disrupting the plasma membrane localization and the resistance of FSP1 to ferroptosis	9
Ubiquitination	NCOA4	-	TRIM7	GBM	Overexpression of the E3 ubiquitin ligase TRIM7 leads to the ubiquitination of NCOA4, inhibiting NCOA4-mediated ferroptosis in GBM.	71, 72
Ubiquitination	GPX4	K48	TRIM25	Pancreatic cancer	TRIM25 is activated by E2 Ubc5 domain, enabling N6F11 to activate the UbcH5b-TRIM25-GPX4 ubiquitination cascade, triggering ferroptosis in cancer cells.	76
Ubiquitination	SLC7A11/xCT	K37	TRIM3	NSCLC	Overexpression of TRIM3 can increase ROS levels and lipid peroxidation in cancer cells, promoting ferroptosis.	77
Ubiquitination	VDAC2/3	-	Nedd4	Melanoma	The E3 ubiquitin ligase Nedd4 degrades the voltage-dependent anion channel VDAC2/3 by binding to the PPxY motif of VDAC2/3 through its WW domain.	78
Ubiquitination	GPX4	-	Nedd4	PD	Nedd4 mediates the ubiquitination of GPX4, disrupting its function and inducing ferroptosis.	79
Ubiquitination	TfR1	-	HUWE1	I/R	TfR1 for ubiquitination and proteasomal degradation, regulating iron metabolism, counteracting abnormal iron accumulation, inhibiting ferroptosis, and potentially mitigating acute liver injury caused by I/R.	80
Ubiquitination	hnRNPA1	-	USP7	GC	USP7 stabilizes hnRNPA1 through deubiquitination, mediating the entry of miR-522 from cancer-associated fibroblasts in the tumor microenvironment into exosomes.	82
Ubiquitination	SCD	-	USP7	GC	The USP7 inhibitor DHPO induces ferroptosis in gastric cancer, and the inhibition of USP7 by DHPO increases the ubiquitination of SCD, accelerating its proteasomal degradation, thereby inhibiting the growth and metastasis of GC.	83
Ubiquitination	GPX4	K48	USP8	Colorectal tumor	USP8 stabilizes GPX4 by removing K48-linked ubiquitination on GPX4, thereby inhibiting ferroptosis and potentially enhancing cancer immunotherapy.	84
Ubiquitination	histone 2A	-	BAP1	Cancer	The tumor suppressor BAP1 reduces the occupancy of H2Aub at the SLC7A11 promoter.	85
Ubiquitination	SLC7A11	K48	SOCS2	HCC	SOCS2 promotes ferroptosis by facilitating the ubiquitination and degradation of SLC7A11, where SLC7A11 polyubiquitination mainly occurs in the form of K48-linked ubiquitin chains.	86
Ubiquitination	Sirt3	-	USP11	Gallbladder cancer	USP11 can directly bind to Sirt3 and deubiquitinate Sirt3 to stabilize Sirt3, inhibiting its degradation. The occurrence of IVDD downregulates the level of Sirt3, upregulates the level of oxidative stress, induces ferroptosis, and exacerbates patient pain.	93
SUMOylation	Nrf2	K110	SENPs	HCC	Enhance Nrf2's activity in scavenging ROS, ultimately enhancing the tolerance of liver cancer cells to oxidative stress.	95
SUMOylation	ACSL4	-	SENP1	Lung cancer	Overexpression of SENP1 inhibits ferroptosis induced by Erastin or cisplatin, reduces the expression of ACSL4, and induces the expression of SLC7A11.	68
SUMOylation	FSP1	K162	SENP3	-	SENP3 interacts with and de-SUMOylation FSP1 at the K162 site, sensitizing macrophages to ferroptosis.	101
SUMOylation	TRIM28/ACSL4	K532	SENP3	Spinal cord injury	The E3 ubiquitin ligase TRIM28 is upregulated in spinal cord injury, catalyzing the conjugation of SUMO3 to lysine 532 of ACSL4.	104
Phosphorylation	ACC	T79	AMPK	-	AMPK mediates the phosphorylation of ACC, inactivating it by phosphorylating the threonine at position 79, which promotes fatty acid oxidation, reduces serum free fatty acids, and subsequently decreases lipid deposition in tissues, improving lipid metabolism.	103
Phosphorylation	BECN1	S90/93/96	AMPK	CRC	AMPK-induced phosphorylation of BECN1 can promote the formation of the BECN1-SLC7A11 complex and LPO.	62
Phosphorylation	AMPK-mTOR	-	SIRT3	Gestational diabetes	The activation of SIRT3 depletes the activation of the AMPK-mTOR pathway and enhances GPX4 levels, thereby inhibiting autophagy and ferroptosis.	107
Phosphorylation	UMPS	S214	AMPK	Cervical cancer	AMPK phosphorylates the S214 site of UMPS and enhances pyrimidine body assembly, thereby promoting DHODH-mediated resistance to ferroptosis.	110
Phosphorylation	ACSL4	-	PKCβII	-	PKCβII, by sensing initial lipid peroxides, amplifies ferroptosis-related LPO through the phosphorylation and activation of ACSL4.	16
Phosphorylation	ACSL4	T624	LHPP	Prostate cancer	LHPP interacts with AKT, thereby inhibiting AKT phosphorylation, which subsequently inhibits the phosphorylation of ACSL4 at the T624 site.	117
Phosphorylation	GPX4	S104	IGF1R-AKT-CKB	HCC	IGF1R signal activates AKT to phosphorylate CKB at the T133 site, reducing its metabolic activity and increasing its binding and phosphorylation with GPX4 at the S104 site.	12
Acetylation	TP53	K98R+3KR	CREBBP	Acute lung injury	CREBBP as a key acetyltransferase for TP53 acetylation, suppresses the expression of SLC7A11 at the transcriptional level in an acute lung injury model.	9, 126
Acetylation	p53	-	SIRT1	kidney stones	The activation of the deacetylase SIRT1 or the triple mutation of p53 induces the deacetylation of p53, inhibits ferroptosis, and alleviates renal fibrosis caused by CaOx crystals.	129
Acetylation	ALOX12	-	CSE/H2S	-	CSE/H2S can attenuate the acetylation of ALOX12 and prevented lipid peroxidation in the membrane.	135
Acetylation	HMGB1	-	HDAC	-	Various oxidative stresses can induce the release of highly acetylated HMGB1 under various pathological conditions, which can induce the occurrence of ferroptosis.	136
Acetylation	Mitochondrial GPX4	-	SIRT3	AKI	The downregulation of SIRT3 may lead to mitochondrial GPX4 acetylation involved in cadmium-induced renal cell ferroptosis.	137
Acetylation	FSP1 mRNA	N4	NAT10	Colon cancer	The N4 acetylation modification of FSP1 mRNA is associated with the inhibition of ferroptosis, and the knockdown of NAT10 significantly increases ferroptosis in colon cancer cells.	138
Acetylation	NNT	K1042	IL-1β	GC	Under IL-1β stimulation, the NNT in cancer cells is acetylated at lysine 1042, inducing the mitochondrial translocation of PCAF, which maintains sufficient iron-sulfur clusters and protects tumor cells from ferroptosis.	145
Methylation	Histone H3	K4	KMT2B	I/R	KMT2B accelerates the RFK by enhancing H3 methylation levels, thereby activating the TNF-α/NOX2 pathway and promoting ferroptosis induced by myocardial I/R.	149
Lipidation	HpETE-PEs	-	15LO1-PEBP1	Asthma	15-LO1 in HAECs forms a 15-LO1-PEBP1 complex with PEBP1, which generates iron-avid HpETE-PEs that lipidate the autophagy protein microtubule LC-3I, forming membrane-bound lipidated LC3-II. This process stimulates the initiation of protective autophagy, allowing cells to escape ferroptosis and the release of mitochondrial DNA.	161
N-myristoylation	FSP1	-	ACSL1	Ovarian cancer	The upregulation of ACSL1 reduces the levels of LPO, thereby enhancing the cells' resistance to ferroptosis. FSP1 as a potential candidate for N-myristoylation, further augments ferroptosis resistance in an ACSL1-enhanced manner.	34
N-myristoylation	FSP1	-	NADPH/NMT2	Neurodegenerative diseases	Exogenous NADPH interacts with NMT2, upregulating N-myristoylated FSP1. NADPH increases the membrane localization of FSP1, enhancing resistance to ferroptosis.	37
Palmitoylation	CD8^+^ T cell	-	CPT1A	Lung cancer	CPT1A identified as a key rate-limiting enzyme in fatty acid oxidation, which acts in concert with l-carnitine from tumor-associated macrophages to drive ferroptosis resistance and CD8^+^ T cell inactivation in lung cancer.	172
Palmitoylation	SLC7A11	Cys327	ZDHHC8	GBM	SLC7A11 undergoes S-palmitoylation, which is catalyzed by ZDHHC8, a member of the PAT, at the Cys327 site, thereby reducing the ubiquitination level of SLC7A11.	18
Palmitoylation	IFNγ signaling pathway	-	-	CRC	IFNGRs and Janus kinase/STAT1 can modulate the IFNγ signaling pathway through palmitoylation. The downregulation of the two subunits of system Xc-, SLC3A2 and SLC7A11, impairs the tumor cells' cystine uptake, promoting LPO and ferroptosis in tumor cells.	174
Palmitoylation	GPX4	Cys66	ZDHHC20	-	The Cys66 residue of GPX4 is palmitoylated by the acyltransferase ZDHHC20, thereby increasing its stability and mitigating ferroptosis. Conversely, inhibition of the depalmitoylase APT2 enhances GPX4 palmitoylation, promoting ferroptosis resistance.	175
Glycosylation	Ferritin heavy chain	Ser179	RSL3	-	RSL3 lead to the removal of O-GlcNAc from serine 179 of the ferritin heavy chain, which increases its binding to the ferritin receptor NCOA4. This modification results in the accumulation of labile iron within the mitochondria.	185
Glycosylation	SLC3A2	-	B3GNT3	PDAC	B3GNT3 can catalyze the glycosylation of SLC3A2, stabilize the SLC3A2 protein, and enhance the interaction between SLC3A2 and xCT.	20
Glycosylation	SREBP1	-	ALG3	Cancer	Inhibition of ALG3 induces defects in post-translational N-linked glycosylation modifications and leads to excessive lipid accumulation in cancer cells through SREBP1-dependent lipogenesis, inducing immunogenic ferroptosis in cancer cells.	187
Lactylation	PCK2	Lys100	KAT8	IRI	Hyperlactatemia exacerbates ferroptosis in hepatic IRI by activating KAT8, which lactylates PCK2 at Lys100, thereby enhancing its kinase activity.	207
Histone Lactylation	Histone H3	K18	p300	Sepsis	Lactate regulates the level of m^6^A modification by promoting the binding of lysine 18 on histone H3 acetylated by lysine acetyltransferase p300 to the METTL3 promoter site.	208

**Table 2:** ** The potential regulatory role of some traditional Chinese medicine components in ferroptosis through protein modification.** Delineates the potential mechanisms by which natural phytochemicals from traditional Chinese medicinal herbs modulate ferroptosis-related proteins through various PTMs in disease treatment.

Modification	Natural Herbs	Targets	Disease	Mechanism	Ref.
Ubiquitination	Curcumin	AR	GBM	AR enhances the drug resistance of GBM to TMZ, and curcumin analog ALZ003-mediated AR ubiquitination causes astrocytoma cells to undergo ferroptosis.	94
SUMOylation	Baicalin	SIRT3	DCM	Baicalin is a bioactive compound with similar cardioprotective effects to the overexpression of SENP1. Sentrin/SENP1 regulates the deSUMOylation process of SIRT3, enhances mitochondrial quality control, prevents cell death, and ultimately improves diabetic cardiomyopathy.	100
Phosphorylation	PrA	ACSL4 and FTH1	DIC	PrA interacts with ACSL4 and FTH1, thereby suppressing ACSL4 phosphorylation and phospholipid peroxidation, and concurrently inhibiting FTH1 autophagic degradation and Fe²⁺ release.	118
Phosphorylation	Curcumin	Sirt1/AKT/FoxO3a	MIRI	Curcumin attenuates autophagy-dependent ferroptosis induced by myocardial ischemia-reperfusion via the Sirt1/AKT/FoxO3a signaling pathway.	108
Phosphorylation	SFN	Nrf2	AKI	SFN intervention can interact with Nrf2 and autophagy to prevent ferroptosis in AKI.	121
Phosphorylation	Bergapten	PI3K	Renal fibrosis	Bergapten, a natural coumarin derivative found in citrus peel, has been reported to inhibit PI3K phosphorylation and indirectly restore GPX4 expression.	120
Acetylation	Paeoniflorin	p53	Brain injury	Paeoniflorin promotes p53 ubiquitination and degradation via the proteasome, inhibits p53 acetylation, reduces its stability, and regulates the SLC7A11-GPX4 pathway to inhibit ferroptosis.	134
Lactylation	Evodiamine	HIF1A histone	Prostate cancer	Evodiamine inhibits the expression of HIF1A histone lactylation in prostate cancer cells, significantly blocking lactate-induced angiogenesis. It further enhances the transcription of the signaling protein Sema3A, suppresses the transcription of PD-L1, and induces ferroptosis through the expression of GPX4.	212

## Abbreviations

GPX4: Glutathione peroxidase 4
ROS: Reactive oxygen species
PTMs: Post-translational modifications
GPI: Glycosyl phosphatidylinositol
PRDX3: Peroxiredoxin 3
FSP1: Ferroptosis suppressor protein 1
DHODH: Dihydroorotate dehydrogenase
GCH1: GTP cyclohydrolase 1
BH4: Tetrahydrobiopterin
GSH: Glutathione
SLC3A2: Solute carrier family 3 member 2
SLC7A11: Solute carrier family 7 member 11
PLOOH: Phospholipid hydroperoxides
PTL: Parthenolide
TNBC: Triple-negative breast cancer
CKB: Creatine kinase B
mTORC2: Mtor complex 2
ACSL1: Acyl-coa Synthetase Long-Chain Family Member 1
CRISPRa: CRISPR-Cas9 gene screening and genome-wide dcas9-based activation screening
IMM: Inner membrane
TMZ: Temozolomide
LOXL3: Lysyl oxidase-like 3
AK2: Adenylate kinase 2
MBOAT1/2: Membrane-bound O-acyltransferase domain containing 1 and 2
PE: Phosphatidylethanolamine
PUFA: Polyunsaturated fatty acid
AA: Arachidonic acid
AdA: Adrenic acid
ALOX: Arachidonate lipoxygenases
PEBP1: PE-binding protein 1
POR: P450 oxidoreductase
MUFAs: Monounsaturated fatty acids
ACSL3: Acyl-coa synthetase long-chain family member 3
DMT1: Divalent metal transporter 1
LIP: Labile iron pool
NCOA4: Nuclear receptor coactivator 4
EAAT3: Excitatory amino acid transporter 3
GCL: Glutamate-cysteine ligase
MAT2A: Methionine adenosyltransferase 2A
DUBs: Deubiquitinases
SMURF2: SMAD-specific E3 ubiquitin ligase 2
GSTP1: Glutathione S-transferase P1
DOX: Doxorubicin
NSCLC: Non-small cell lung cancer
PD: Parkinson's disease
I/R: Ischemia-reperfusion
USP7: Ubiquitin-specific protease 7
hnRNPA1: Heterogeneous nuclear ribonucleoprotein A1
SCD: Stearoyl-coa desaturase
USP8: Ubiquitin-specific protease 8
BAP1: BRCA1-associated protein 1
HCC: Hepatocellular carcinoma
CCA: Cholangiocarcinoma
SHARPIN: Shank-associated RH domain interacting protein
MDA: Malondialdehyde
NO: Nitric oxide
CRC: Colorectal cancer
IVDD: Intervertebral disc degeneration
USP11: Ubiquitin-specific protease 11
SIRT3: Sirtuin 3
PK: Protein kinases
PPase: Protein phosphatases
AMPK: AMP-activated protein kinase
ACC: Acetyl-coa carboxylase
PRKCQ: Protein kinase C theta
HPCAL1: Hippocampal calcium-binding protein-like 1
CDH2: Cadherin 2
GLP-1: Glucagon-like peptide-1
MTOR: Mechanistic target of rapamycin kinase
DHO: Dihydroorotate
PrA: Protosappanin A
IGF1R: Insulin-like growth factor 1 receptor
OXA: Oxaliplatin
Nrf2: Nuclear factor erythroid 2-related factor 2
SFN: Sulforaphane
CREBBP/CBP: CREB-binding protein
SIRT1: Sirtuin 1
CTH/CSE: Cystathionine γ-lyase
GINS4: GINS complex subunit 4
LUAD: Lung adenocarcinoma
CaOx: Calcium oxalate
NAMPT: Nicotinamide phosphoribosyltransferase
HDAC: Histone deacetylase
HAT: Histone acetyltransferase
AKI: Acute kidney injury
NAT10: N-acetyltransferase 10
HMGA2: High mobility group AT-hook 2
CAP: Cold atmospheric plasma
IL-1β: Interleukin-1β
HMT: Histone methyltransferases
HKMTs: Histone lysine methyltransferases
PRMTs: Protein arginine methyltransferases
KMT2B: Lysine-specific methyltransferase 2B
PRMT7: Protein arginine methyltransferase 7
PRMT4: Protein arginine methyltransferase 4
LC-3: Light chain protein-3
HAECs: Human airway epithelial cells
LC-3I: Light chain protein-3I
RPE: Retinal pigment epithelial
LCN2: Lipocalin 2
GBM: Glioblastoma multiforme
MYR: N-myristoylation
NMT: N-myristoyltransferase
ZDHHC: Zinc finger, aspartate-histidine-histidine-cysteine
CPT1A: Carnitine palmitoyltransferase 1A
PAT: Protein acyltransferase
IFNγ: Interferon-gamma
IFNGRs: Interferon-gamma receptor
CS: Cigarette smoke
RPE: Retinal pigment epithelial
ER: Estrogen receptor
SREBP1: Sterol regulatory element-binding protein
GSNO: S-nitrosoglutathione
RNS: Reactive nitrogen species
ACLY: ATP citrate lyase
HDACi: Histone deacetylase inhibitors
TEC: Tectorigenin
MAP4K4: Mitogen-activated protein kinase kinase kinase kinase 4
SNO-Drp1: S-nitrosylation of Drp1
EPHX1: Microsomal epoxide hydrolase 1
MALT1: Mucosa-associated lymphoid tissue lymphoma translocation protein 1
PCK2: Phosphoenolpyruvate carboxykinase 2
